# The *Sclerotinia sclerotiorum* Mating Type Locus (*MAT*) Contains a 3.6-kb Region That Is Inverted in Every Meiotic Generation

**DOI:** 10.1371/journal.pone.0056895

**Published:** 2013-02-15

**Authors:** Periasamy Chitrampalam, Patrik Inderbitzin, Karunakaran Maruthachalam, Bo-Ming Wu, Krishna V. Subbarao

**Affiliations:** Department of Plant Pathology, University of California Davis, Davis, California, United States of America; Soonchunhyang University, Republic of Korea

## Abstract

*Sclerotinia sclerotiorum* is a fungal plant pathogen and the causal agent of lettuce drop, an economically important disease of California lettuce. The structure of the *S. sclerotiorum* mating type locus *MAT* has previously been reported and consists of two idiomorphs that are fused end-to-end as in other homothallics. We investigated the diversity of *S. sclerotiorum MAT* using a total of 283 isolates from multiple hosts and locations, and identified a novel *MAT* allele that differed by a 3.6-kb inversion and was designated Inv+, as opposed to the previously known *S. sclerotiorum MAT* that lacked the inversion and was Inv-. The inversion affected three of the four *MAT* genes: *MAT1-2-1* and *MAT1-2-4* were inverted and *MAT1-1-1* was truncated at the 3’-end. Expression of *MAT* genes differed between Inv+ and Inv- isolates. In Inv+ isolates, only one of the three *MAT1-2-1* transcript variants of Inv- isolates was detected, and the alpha1 domain of Inv+ *MAT1-1-1* transcripts was truncated. Both Inv- and Inv+ isolates were self-fertile, and the inversion segregated in a 1∶1 ratio regardless of whether the parent was Inv- or Inv+. This suggested the involvement of a highly regulated process in maintaining equal proportions of Inv- and Inv+, likely associated with the sexual state. The *MAT* inversion region, defined as the 3.6-kb *MAT* inversion in Inv+ isolates and the homologous region of Inv- isolates, was flanked by a 250-bp inverted repeat on either side. The 250-bp inverted repeat was a partial *MAT1-1-1* that through mediation of loop formation and crossing over, may be involved in the inversion process. Inv+ isolates were widespread, and in California and Nebraska constituted half of the isolates examined. We speculate that a similar inversion region may be involved in mating type switching in the filamentous ascomycetes *Chromocrea spinulosa, Sclerotinia trifoliorum* and in certain *Ceratocystis* species.

## Introduction

*Sclerotinia sclerotiorum* is a filamentous ascomycete in the *Sclerotiniaceae* (Pezizomycotina) and a necrotrophic pathogen of more than 400 hosts worldwide, including many important agricultural crops [1], [2]. In California, the biggest lettuce producer in the United States, *S. sclerotiorum* is a causal agent of lettuce drop [3] that reduces overall annual lettuce yield by 15%, and losses in individual fields commonly amount to up to 60% [4].

*Sclerotinia sclerotiorum* is very durable and survives in the soil in absence of a host for one or more years [5], [6]. The survival structures are known as sclerotia that are clusters of cells surrounded by a melanized protective layer. New infections are initiated when sclerotia germinate to form hyphae, or when ascospores are released from fruiting bodies, known as apothecia that emerge from sclerotia [7]. Ascospores result from selfing [8] or outcrossing by heterokaryon formation and recombination [9]. Microconidia are formed and may play a role in fertilization [10].

In ascomycetes, mating system is determined by the mating type locus *MAT* that encodes transcription factors that regulate downstream gene expression [11]. There are two major mating systems which are heterothallism that is equivalent to obligate outcrossing, and homothallism that consists of selfing and outcrossing [12]. In heterothallic species, isolates generally carry one of two versions of *MAT*, and only isolates that differ at *MAT* are sexually compatible. In homothallic species, there is only one version of *MAT* that generally consists of all the *MAT* genes of heterothallic relatives, but assembled at a single locus [13]. The two versions of *MAT* in heterothallics are referred to as idiomorphs instead of alleles, because overall DNA sequence homology between idiomorphs is absent [14], [15].

The nomenclature of *MAT* loci, idiomorphs and *MAT* genes is based on alpha1 and high mobility group (HMG) domain genes that encode transcription factors [16]. Most filamentous ascomycetes have a single *MAT* locus [12]. ‘*MAT1-1’* refers to the idiomorph that contains an alpha1 gene, and ‘*MAT1-2*’ refers to the idiomorph that contains an HMG domain gene. All heterothallic ascomycete species examined in detail contain one *MAT1-1* and one *MAT1-2* idiomorph [12]. The alpha1 gene present at *MAT1-1* is designated ‘*MAT1-1-1’,* and the HMG box gene at *MAT1-2* is referred to as ‘*MAT1-2-1’.* Other genes are present at *MAT* depending on the species, and names for new genes are created taking idiomorphs and previously discovered genes into account. For instance, the second gene discovered at *MAT1-1* was designated *MAT1-1-2,* the third gene *MAT1-1-3,* the fourth *MAT1-1-4,* and so on, and similarly at *MAT1-2,* with genes *MAT1-2-2, MAT1-2-3, MAT1-2-4,* and so on [16].

*Sclerotinia sclerotiorum* is a typical homothallic. Selfing [8] and outcrossing [9] have been demonstrated, and the *MAT1-1* and *MAT1-2* idiomorphs are fused end-to-end at *MAT* [17]. *Sclerotinia sclerotiorum MAT* thus consists of four genes, *MAT1-1-1, MAT1-1-5, MAT1-2-1* and *MAT1-2-4* and the same genes are present in the close relative and heterothallic *Botrytis cinerea,* but in a heterothallic *MAT* arrangement where *MAT1-1* individuals contain *MAT1-1-1* and *MAT1-1-5,* and *MAT1-2* individuals *MAT1-2-1* and *MAT1-2-4* [17].

Whereas most filamentous ascomycete species are either heterothallic or homothallic, there are examples of mating type switching that occurs within a single generation, from heterothallism to homothallism [18], [19], [20], and vice versa [21], [22], [23]. Mating systems also change over larger evolutionary time scales, and in general homothallic species evolve from heterothallic ancestors [24], [25], [26], [27].

The role of *MAT* as master regulator of downstream gene expression has been well studied in some ascomycetes including *S. cerevisiae* [28], but little is known about *MAT* regulated genes in filamentous ascomycetes. *MAT* target genes in filamentous ascomycetes include pheromone and pheromone receptors that play important roles in recognition of mating partners [29], [30], [31], and possibly in nuclear pairing following fertilization [32], [33], [34], [35], and genes that are involved in heterokaryon incompatibility [36]. But the majority of genes under *MAT* regulation appear to have functions that are not directly related to mating [31], [37], [38], [39]. These pleiotropic capabilities of *MAT* may explain the morphological changes that correlate with mating system in species where mating type switching has been documented [21], [23], [40].

In this study, we examined the mating type loci of 283 *S. sclerotiorum* isolates from lettuce in California and other states and hosts, and found that *MAT* contained a 3.6-kb region that is inverted between generations which correlates with changes in *MAT* gene expression. We show that the fused *S. sclerotiorum MAT* arrangement most likely evolved from heterothallic ancestors, and speculate whether a *MAT* inversion similar to the one in *S. sclerotiorum* may be responsible for mating type switching in several other filamentous ascomycetes.

## Results

### *Sclerotinia sclerotiorum* MAT locus

**Figure 1:**
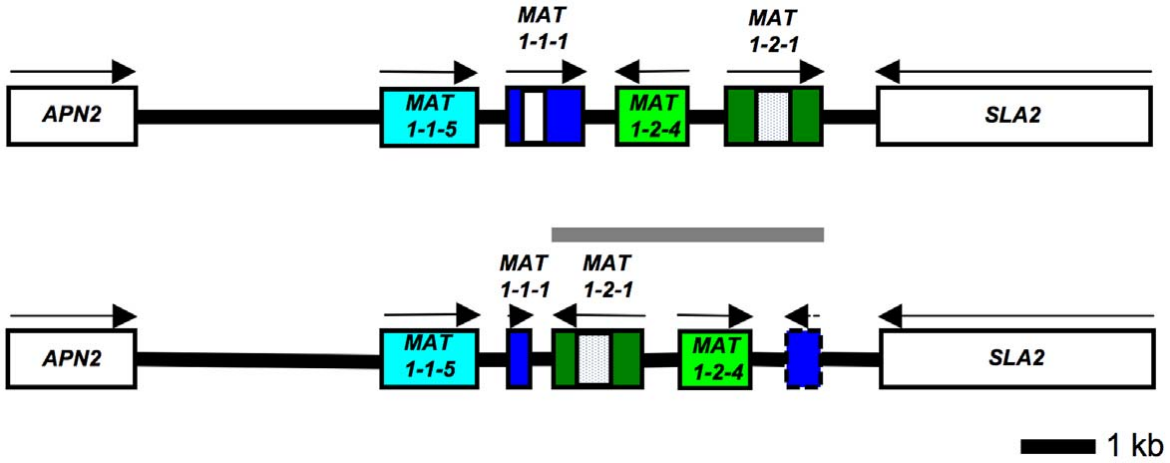
Maps of *Sclerotinia sclerotiorum* mating type alleles that differ by a 3.6-kb inversion. Inv- (top) and Inv+ (bottom) *MAT* alleles are shown, the 3.6-kb *MAT* inversion region is indicated by a gray horizontal line. Genes are colored boxes, white and dotted boxes within genes correspond to alpha1 and HMG domains, respectively, flanking genes are white boxes, directions of transcription are indicated by arrows, gene names are inside or by the boxes. Dashed box and arrow represent *MAT1-1-1* 3’-fragment lacking an in frame start codon. Diagrams are to scale. The Inv+ alpha1 box is truncated after 45 bp and is not illustrated, for details see text.

DNA sequencing coverage of three complete *MAT* regions was generated for *S. sclerotiorum* strains 44Ba1, 44Ba12 and 44Ba18 that are listed in Table S1. The DNA sequences measured 12,145 bp, 12,218 bp and 12,218 bp, respectively, and were submitted to GenBank (Accessions JQ815883, JQ815884, and JQ815885). The previously sequenced *S. sclerotiorum* strain 1980 *MAT* region from the recently completed genome sequencing project [17], was more than 98% similar overall across coding and non-coding regions to homologous regions of *S. sclerotiorum* strains 44Ba1, 44Ba12 and 44Ba18 (Table 1). The *S. sclerotiorum* strain 44Ba1 *MAT* region contained the same ORFs as *S. sclerotiorum* strain 1980 [17], notably *MAT1-1-5*, *MAT1-1-1*, *MAT1-2-4* and *MAT1-2-1*, flanked by *APN2* and *SLA2*, whereas *S. sclerotiorum* strains 44Ba12 and 44Ba18 differed by a 3.6-kb inversion that extended from *MAT1-1-1* to *MAT1-2-1*, and resulted in the inversion of *MAT1-2-1* and *MAT1-2-4*, and the truncation of *MAT1-1-1* at the 3’-end (Figure 1; Alignment S1). Following the genetic nomenclature of plant pathogenic fungi on the designation of phenotypes [41], isolates with the *MAT* inversion were designated Inv+, and isolates without the inversion Inv-. The 3.6-kb *MAT* inversion in Inv+ isolates, and the homologous region in Inv- isolates, were referred to as the *MAT* inversion region.

**Table 1:** Mating type gene statistics of *Sclerotinia sclerotiorum* strains 44Ba1, 44Ba12. and 44Ba18 in relation to *S. sclerotiorum* strain 1980 and *Botrytis cinerea* [17].

Gene name	Species	Strain identifier	GenBank / Broad Institute accession number	Gene length, bp^A^	Similarity, %	Intron length, bp (position)^A^			Coding sequence, bp^A^	Protein length, aa^A^
						Intron 1^B^	Intron 2^B^	Intron 3^B^		
*MAT1-1-1*	*S. sclerotiorum*	1980	XM_001594147.1/ SS1G04004	826	NA	-	50 (761–809)	-	777	259
	*S. sclerotiorum*	44Ba1	JQ815883	1106	99.6	49^C^ (224–272)	50 (1040–1089)	-	1005	335
	*S. sclerotiorum*	44Ba12	JQ815884	354	98.6	Absent^C^	-	-	354	117
	*S. sclerotiorum*	44Ba18	JQ815885	354	98.6	Absent^C^	-	-	354	117
	*B. cinerea*	B05.10	XM_001546388.1/ BC1G_15148	1161	72	50 (260–309)	49 (1096–1144)	-	1062	354
*MAT1-1-5*	*S. sclerotiorum*	1980	XM_001594146.1/SS1G04003	1303	NA	49 (281–329)	64 (878–941)	59 (969–1027)	1131	377
	*S. sclerotiorum*	44Ba1	JQ815883	1303	99.7	49 (281–329)	64 (878–941)	59 (969–1027)	1131	377
	*S. sclerotiorum*	44Ba12	JQ815884	1303	99.8	49 (281–329)	64 (878–941)	59 (969–1027)	1131	377
	*S. sclerotiorum*	44Ba18	JQ815885	1303	99.8	49 (281–329)	64 (878–941)	59 (969–1027)	1131	377
	*B. cinerea*	B05.10	XM_001546387.1/BC1G_15147.1	1301	52.4	49 (281–329)	62 (879–940)	59 (968–1026)	1131	377
*MAT1-2-1*	*S. sclerotiorum*	1980	XM_001594149.1	1289	NA	50 (283–332)	54 (703–756)	-	1185	395
	*S. sclerotiorum*	44Ba1^D^	JQ815883	318	99.7	Absent^C^	Absent^C^	-	318	105
	*S. sclerotiorum*	44Ba1^D^	JQ815883	1289	99.8	50^C^ (283–332)	54^C^ (703–756)	-	1185	395
	*S. sclerotiorum*	44Ba1^D^	JQ815883	318	99.7	Absent^C^	Absent^C^	-	318	105
	*S. sclerotiorum*	44Ba12	JQ815884	1289	99.8	49^B^ (282–330)	54^B^ (703–756)	-	1185	395
	*S. sclerotiorum*	44Ba18	JQ815885	1289	99.8	49^C^ (282–330)	54^C^ (703–756)	-	1185	395
	*B. cinerea*	T4	FQ790352.1	1248	74	49 (283–332)	55 (706–760)	-	1143	380
*MAT1-2-4*	*S. sclerotiorum*	1980	XM_001594148.1/ SS1G04005	944	NA	49 (171–219)	48 (459–506)	-	846	282
	*S. sclerotiorum*	44Ba1	JQ815883	944	99.5	49 (171–219)	48 (459–506)	-	846	282
	*S. sclerotiorum*	44Ba12	JQ815884	944	99.6	49 (171–219)	48 (459–506)	-	846	282
	*S. sclerotiorum*	44Ba18	JQ815885	944	99.6	49 (171–219)	48 (459–506)	-	846	282
	*B. cinerea*	T4	FQ790352.1	1517	72	48 (192–239)	48 (480–527)	190 (680–869)	1230	409

A Gene lengths, intron lengths and positions, transcript lengths and amino acid translations for *S. sclerotiorum* strains 44Ba1, 44Ba12 and 44Ba18 were conceptually deduced by comparison to *S. sclerotiorum* strain 1980 [17] unless indicated otherwise.

B Intron 1, 2 and 3 refer to the relative positions of the introns in each gene from 5’- to 3’-end.

C Confirmed by RT-PCR and DNA sequencing.

D More than one transcript was obtained and all were listed by transcript size, see Figures 6, 7.

**Figure 2:**
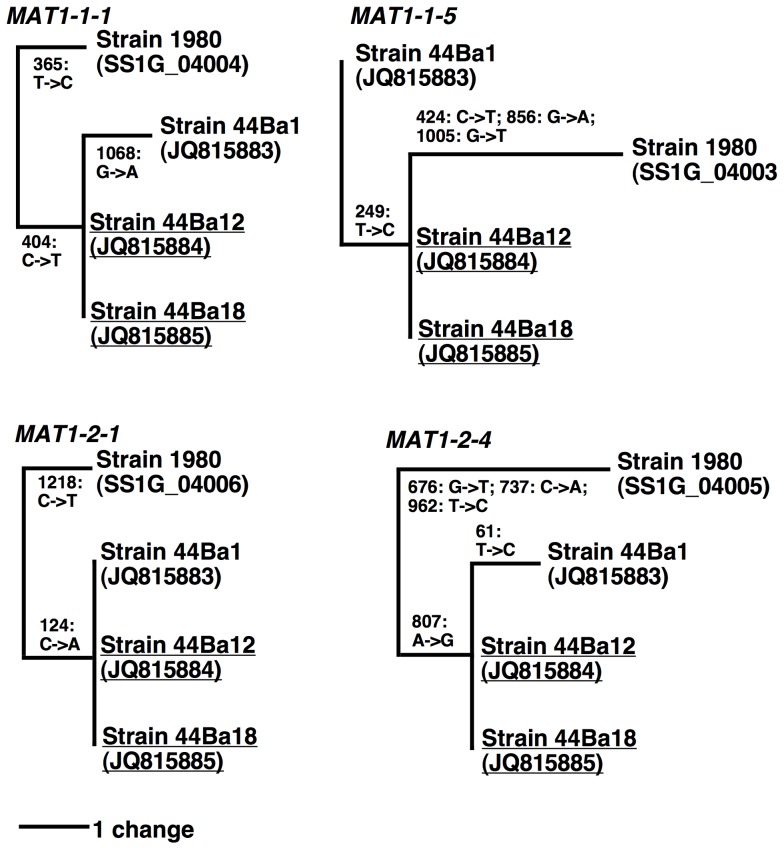
Most parsimonious trees obtained from *Sclerotinia sclerotiorum MAT1-1-1, MAT1-1-5, MAT1-2-1* and *MAT1-2-4* single locus datasets (Alignments S6, S7, S8, S9). The trees were rooted with *B. cinerea* which is not shown, numbers refer to alignment positions. Nucleotide substitutions along the branches are given for *S. sclerotiorum*. Accession numbers are shown for all sequences following the strain identifiers, for *B. cinerea* the sequence accession numbers are BC1G_15148 (*MAT1-1-1*), BC1G_15147 (*MAT1-1-5*), FQ790352 (*MAT1-2-1*), FQ790352 (*MAT1-2-4*).

There was little variation among the homologous *MAT* regions of the *S. sclerotiorum* isolates. *Sclerotinia sclerotiorum* strains 44Ba12 and 44Ba18 had identical sequences, and among the four *MAT* ORFs, *MAT1-2-4* was most variable, and *MAT1-2-1* was most conserved (Table S2). Phylogenetic analyses showed that irrespective of *MAT* gene, *S. sclerotiorum* strains 1980, 44Ba1, 44Ba12 and 44Ba18 were monophyletic (Figure 2). Due to the low number of parsimony informative characters (Table S3), branch supports were not evaluated. More details on the phylogenetic analyses are given in Table S3.

The non-coding regions of *S. sclerotiorum MAT* differed most notably by insertions and deletions. With respect to *S. sclerotiorum* strain 1980, two deletions of 152 and 26 bp, were present in *S. sclerotiorum* strains 44Ba1, 44Ba12 and 44Ba18 between *APN2* and *MAT1-1-5*. Additionally, strain 44Ba1 included an 11 bp insertion between *MAT1-2-4* and *MAT1-2-1* (Table S4).

Comparisons between the mating type genes of *S. sclerotiorum* generated in this study to the closely related *Botrytis cinerea* [17] revealed 52 to 74% nucleotide identity depending on the *MAT* gene (Table 1). Among the four mating type genes, *MAT1-1-5* was the most divergent with the lowest identity (52%), the remaining three *MAT* genes were more than 71% identical to *B. cinerea* homologs. Except for *MAT1-2-4* where *B. cinerea* had an extra intron, intron numbers were conserved between *S. sclerotiorum* and *B. cinerea* (Table 1).

**Figure 3:**
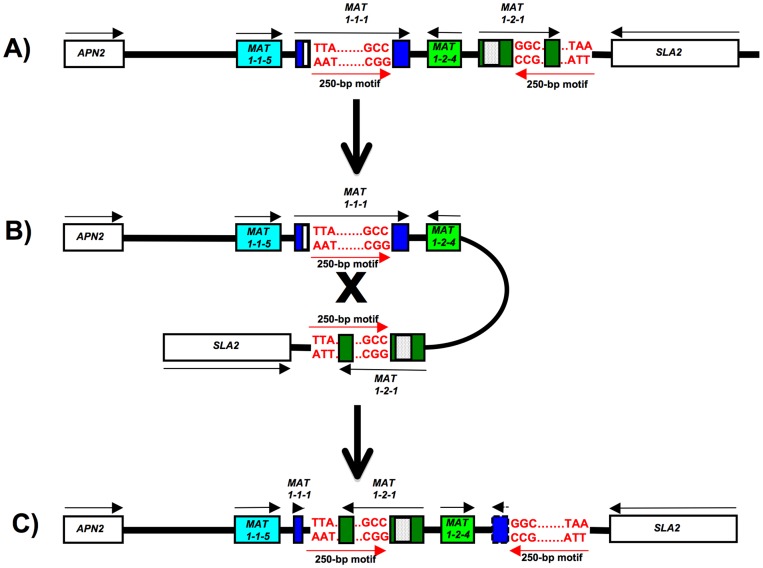
Inversion of the 3.6-kb *MAT* inversion region in *Sclerotinia sclerotiorum* by means of crossing over between the two 250-bp motifs. Genes are colored boxes, white and dotted boxes within genes correspond to alpha1 and HMG domains, respectively, flanking genes are white boxes, directions of transcription are indicated by black arrows, gene names are inside or by the boxes, red arrows mark the orientations of the 250-bp motifs for which the 5’- and 3’-end sequence is given. Dashed box and arrow represent *MAT1-1-1* 3’-fragment lacking an in frame start codon. 3A) *MAT* region of *S. sclerotiorum* Inv- isolates. 3B) Crossing over between the two 250-bp motifs of a *S. sclerotiorum* Inv- isolate gives rise to 3C), the inversion of *S. sclerotiorum* Inv+ isolates. Figures are not to scale. The Inv+ alpha1 box is truncated after 45 bp and is not illustrated, for details see text.

**Figure 4:**
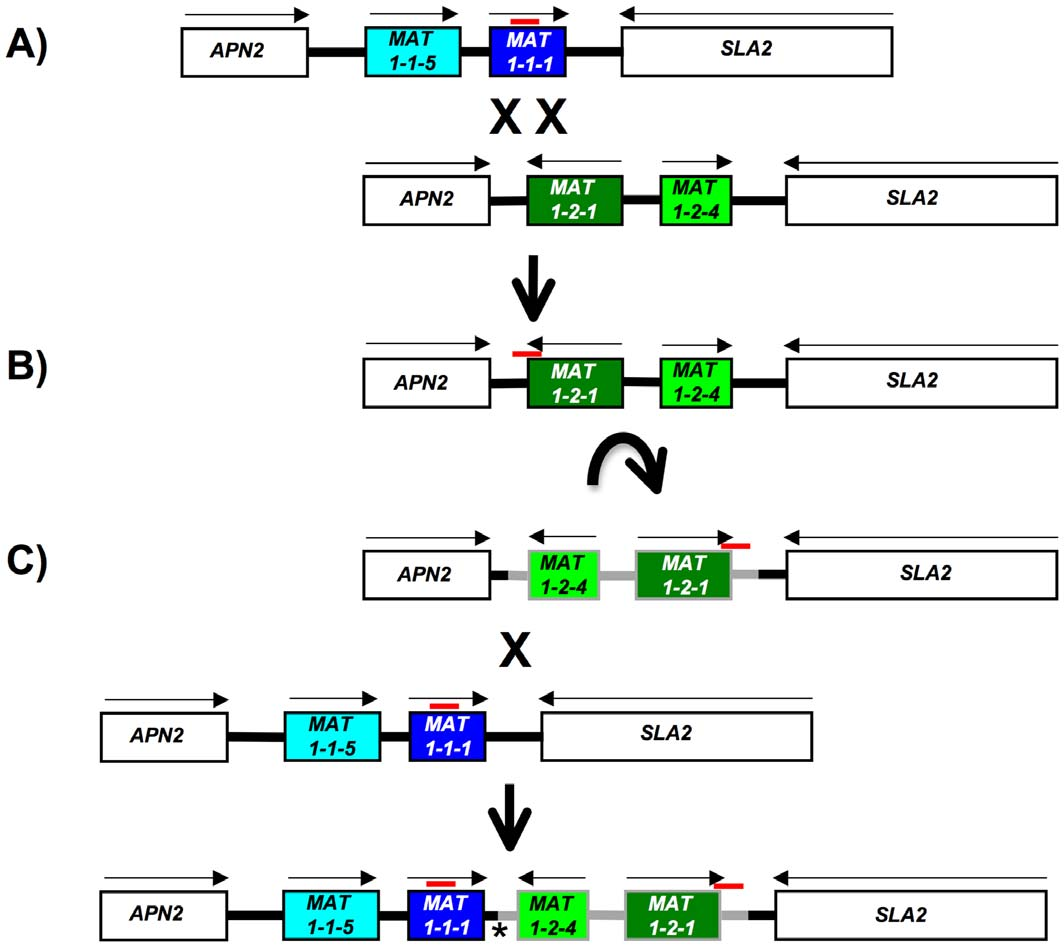
Evolutionary origin of the *MAT* locus in *Sclerotinia sclerotiorum* Inv- isolates from hypothetical ancestors by means of a double crossing over, an inversion and a single crossing over. Genes are boxes, directions of transcription are indicated by arrows, gene names are inside the boxes, positions of the 250-bp motif are indicated by red horizontal lines, crossing overs are marked by ‘X’, the inversion by a curved arrow. 4A) A double crossing over between ancestral *MAT1-1* (blue) and *MAT1-2* (green) transferred a 250-bp fragment, the ‘250-bp motif’, from *MAT1-1-1* to *MAT1-2-1* and flanking region. 4B) A subsequent inversion (gray) in the ancestral *MAT1-2* (green), followed by a crossing over, 4C) with an ancestral *MAT1-1* (blue), results in the *MAT* arrangement in *S. sclerotiorum* Inv- isolates. The asterisk indicates the location of the DNA sequence alignment provided in Figure 5.

Comparison between the flanks of the *MAT* inversion region in the *S. sclerotiorum* Inv+ strains 44Ba12 and 44Ba18, to the homologous regions of the *S. sclerotiorum* Inv- strain 44Ba1, revealed the presence of a 250-bp inverted repeat, referred to as the 250-bp motif, on either side of the *MAT* inversion region. The *MAT1-1-5* proximal 250-bp motif was part of *MAT1-1-1,* and the *MAT1-1-5* distal 250-bp motif was inverted, and overlapped with the 3’-end of *MAT1-2-1* (Figure 3A; Alignment S1). The DNA sequences of the two motifs were identical. Since the 250-bp motif was absent in *B. cinerea MAT1-2-1,* but present in *MAT1-1-1* of both *B. cinerea* and *S. homoeocarpa* (Table S5; Alignment S2), this indicated that the 250-bp motif is a partial *MAT1-1-1* that was integrated into *MAT1-2-1* in an ancestor of *S. sclerotiorum,* possibly through a double crossing over (Figure 4A). The 250-bp motif consisted of 60% AT which is similar to the *S. sclerotiorum* strain 1980 genome overall AT content [17], lacked repeats, and did not match any transposon sequences in GenBank.

**Figure 5:**
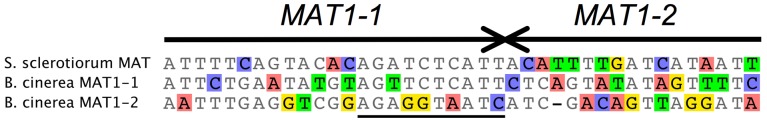
Proposed *MAT1-1 – MAT1-2* border in *S. sclerotiorum MAT* inferred from an alignment of *S. sclerotiorum MAT*, *B. cinerea MAT1-1* and *MAT1-2.* The location of the alignment reproduced above is indicated in Figure 4 by an asterisk, for entire alignment see Alignment S3. *Sclerotinia sclerotiorum* strain 44Ba1 *MAT* (GenBank Accession JQ815883) and *B. cinerea MAT1-1* (AAID01003685) were alignable up to 248 bp downstream of *S. sclerotiorum MAT1-1-1* as indicated by the top left arrow, homology ceases thereafter. The site where a crossover between ancestral *MAT1-1* and *MAT1-2* represented here by *B. cinerea MAT1-1* and *MAT1-2*, might have occurred, is underlined. The border and crossover site positions are tentative, since *S. sclerotiorum MAT* and *B. cinerea MAT1-2* (reverse complement of FQ790352) were too divergent to be aligned in the *MAT1-2-4* downstream region (Figure 1), the top right arrow demarcates the *MAT1-2* region in *S. sclerotiorum* based on lack of homology to *B. cinerea MAT1-1,* not based on homology to *B. cinerea MAT1-2.*

In order to identify the *S. sclerotiorum MAT1-1-1 – MAT1-2-1* fusion junction, we aligned *S. sclerotiorum MAT* with homologous *B. cinerea MAT1-1* and *MAT1-2* regions (Alignment S3), and found that the fusion of *MAT1-1* and *MAT1-2* most likely occurred in a region of 10 bp encompassing nucleotides 239 to 248 downstream of *S. sclerotiorum MAT1-1-1.* This is because DNA sequence homology between *B. cinerea* and *S. sclerotiorum MAT1-1* idiomorphs extended 248 nucleotides downstream of *S. sclerotiorum MAT1-1-1*, and among the ten nucleotides in positions 239 to 248 downstream of *S. sclerotiorum MAT1-1-1,* five were identical between *B. cinerea MAT1-1*, *S. sclerotiorum MAT1-1* and *B. cinerea MAT1-2* (Figure 5; Alignment S3).

### *MAT* gene expression analyses

**Figure 6:**
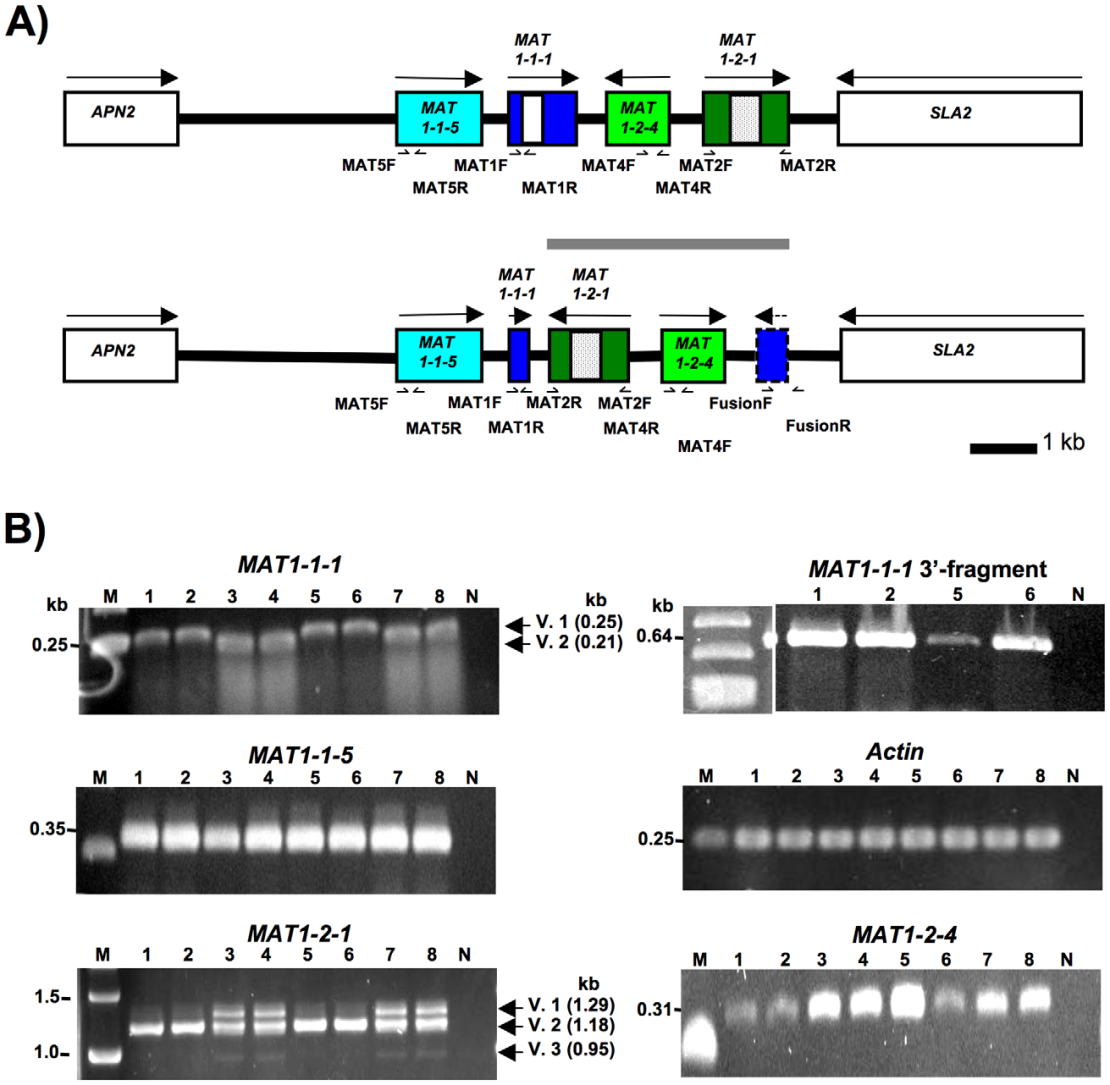
Gene expression in *Sclerotinia sclerotiorum* Inv- and Inv+ isolates. 6A) Positions of RT-PCR primers on *S. sclerotiorum* Inv- (top) and Inv+ (bottom) *MAT* loci, the *MAT* inversion region is indicated by a gray horizontal line. Genes are colored boxes, white and dotted boxes within genes correspond to alpha1 and HMG domains, respectively, flanking genes are white boxes, directions of transcription are indicated by arrows, gene names are inside or by the boxes. Dashed box and arrow represent *MAT1-1-1* 3’-fragment lacking an in frame start codon. Diagrams are to scale. The Inv+ alpha1 box is truncated after 45 bp and is not illustrated, for details see text. 6B) Gels with RT-PCR results for all *MAT* genes, the *MAT1-1-1* 3’-fragment and an actin control, for all eight *S. sclerotiorum* strains 1B331-1 – 1B331-8 representing an ordered tetrad. Gene names are indicated above the gels. Lane numbers refer to *S. sclerotiorum* strain identifiers 1B331-1– 1B331-8. The letters M and N indicate size markers and negative controls, respectively. Band sizes are indicated on the left of each gel. For *MAT1-1-1* and *MAT1-2-1,* the sizes of the transcript variants are indicated on the right of the gels.

**Figure 7:**
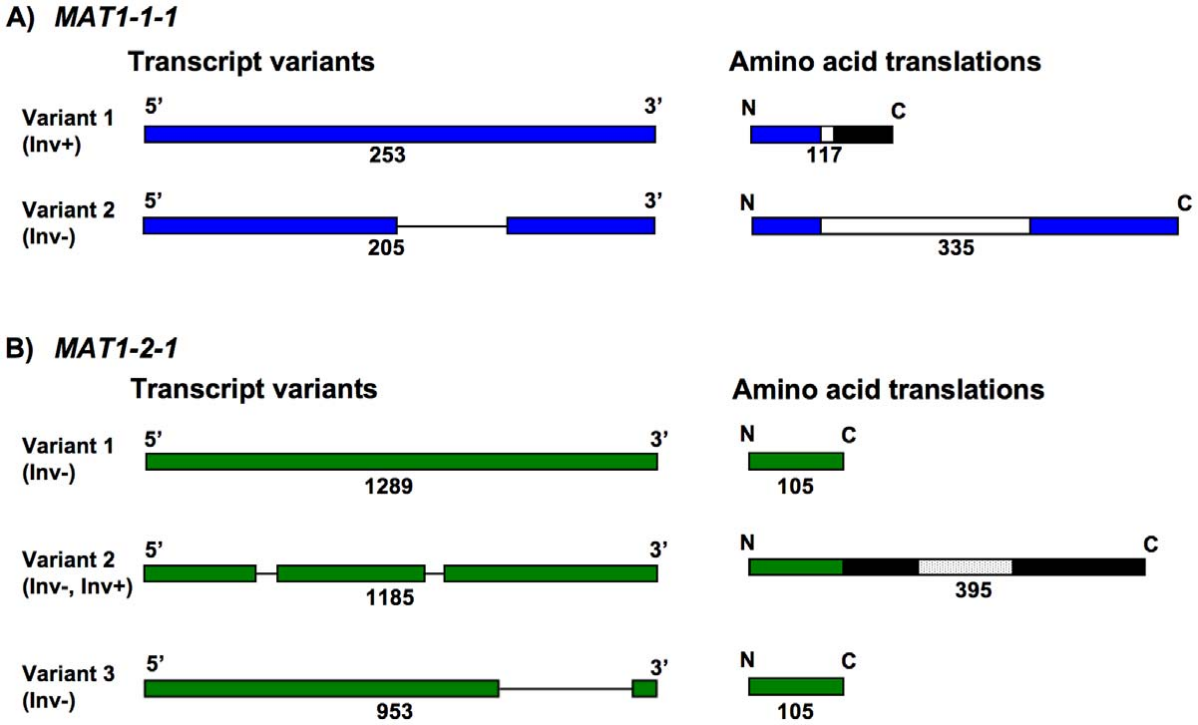
Alignment of *MAT1-1-1* (top) and *MAT1-2-1* (bottom) transcript variants (left) and deduced protein variants (right). Transcript variants are colored boxes, black lines between the boxes represent alignment gaps with respect to other variants, the 5’- and 3’-ends are indicated, variant lengths are given underneath the boxes in base pairs, variant designation and presence in *S. sclerotiorum* Inv- and Inv+ isolates is indicated on the left. Protein variants are boxes, N and C terminals are indicated, white boxes mark alpha1 and dotted boxes mark HMG domains, black boxes represent unalignable regions, variant designation and presence in *S. sclerotiorum* Inv- and Inv+ isolates is indicated on the left. Lengths of protein variants are given underneath the boxes in residues. Protein variants were deduced conceptually taking transcript variants into account. Diagrams are not to scale. 7A) Schematic alignments of *MAT1-1-1* transcript variants from Figure 6B and the corresponding inferred MAT1-1-1 variants. Only transcript variant 2 of *S. sclerotiorum* Inv- isolates implies the presence of a complete alpha1 box in MAT1-1-1. 7B) Schematic alignments of *MAT1-2-1* transcript variants from Figure 6B and the corresponding inferred MAT1-2-1 variants. Only transcript variant 2 of *S. sclerotiorum* Inv- and Inv+ isolates contains an HMG box.

RT-PCR was performed for all four *MAT* genes in the eight *S. sclerotiorum* strains 1B331-1 – 1B331-8 that represented an ordered tetrad. Our results agreed with previous studies with respect to gene boundaries and intron positions [17], with the following exceptions. *MAT1-1-1* was extended by 280 bp at the 5’-end and contained an additional, 49-bp intron. This conclusion was based on the fact that we obtained an RT-PCR product (Figure 6) with a forward primer situated approximately 200 bp upstream of the previously predicted start codon [17], and a *MAT1-1-1* internal reverse primer (Figure 6). Since an in-frame start codon was present 280 bp upstream of the earlier predicted start codon, conceptual translation indicated that *MAT1-1-1* measured at least 1106 bp and contained an additional, 49-bp intron, 223 bp from the 5’-end. The 49-bp intron was spliced in Inv- isolates, but not in Inv+ isolates, resulting in a frame shift, a premature stop codon and a truncated *MAT1-1-1* measuring 354 bp (Figure 7). This is opposed to the 1106 bp-*MAT1-1-1* in Inv- isolates (Table 1). The MAT1-1-1 alpha1 domain in Inv+ isolates was thus shortened to 15 residues, from the 197 residues in Inv- isolates (Figure 7).

A further difference between *MAT* loci was the presence of three *MAT1-2-1* transcript variants in Inv- isolates, only one of the variants was detected in Inv+ isolates (Figure 6). The first variant was unspliced, the second variant was spliced as *MAT1-2-1* in *S. sclerotiorum* strain 1980 [17], and the third variant was spliced differently from the other two variants (Figure 7). Assuming identical start codons for all variants, conceptual translation indicated that variants 1 and 3 encoded identical proteins measuring 105 aa in length and lacking known functional domains, whereas variant 2, the only variant detected in both Inv- and Inv+ isolates, encoded a 395 aa protein containing an HMG domain.

The 3’-fragment of *MAT1-1-1* was also expressed despite the absence of an in-frame start codon (Figure 6) and did not contain any known functional domains.

### *MAT* locus copy number

**Figure 8:**
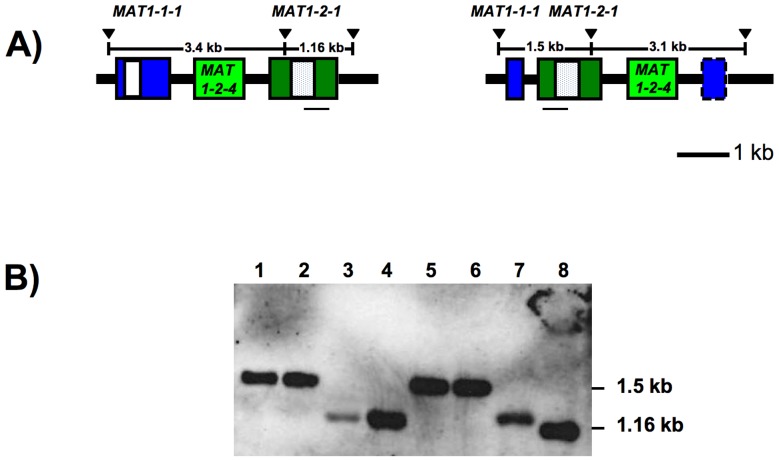
Analysis of *MAT1-2-1* copy number using Southern blotting. 8A) Gene diagrams of *Sclerotinia sclerotiorum* Inv- (left) and Inv+ (right) *MAT* loci illustrating the positions of the Southern probe with respect to the restriction sites used for Southern analyses. Boxes represent genes, white and dotted boxes correspond to alpha1 and HMG domains, respectively, dashed box represents *MAT1-1-1* 3’-fragment lacking an in frame start codon. Gene names are indicated above or within the boxes. The positions of the *Bsa*HI restriction sites (black triangles) and the distances between the *Bsa*HI sites are indicated above the boxes. The position of the Southern probe is marked by a horizontal black line beneath *MAT1-2-1.* The Inv+ alpha1 box is truncated after 45 bp and is not illustrated, for details see text. 8B) Southern blot of *Bsa*HI-digested genomic DNA visualized with the digoxigenin-labeled *MAT1-2-1* specific probe. Wells 1 - 8 correspond to *S. sclerotiorum* strains 1B331-1 – 1B331-8 that represent an ordered tetrad, band sizes are indicated on the right. Lanes 1, 2, 5 and 6 have the pattern reflective of an Inv+ *MAT* locus, lanes 3, 4, 7 and 8 have the pattern expected for an Inv- *MAT* locus.

In order to assess whether the *S. sclerotiorum MAT* locus was single- or multi-copy, Southern hybridization was performed with a *MAT1-2-1* probe and all eight *S. sclerotiorum* strains 1B331-1 – 1B331-8 of the ordered tetrad. *MAT1-2-1* was targeted due to the detection of three transcript variants differing in length, and thus potentially derived from more than one *MAT* region. The results obtained were consistent with the presence of a single *MAT* locus, as all isolates had only one Southern band, diagnostic of the presence or absence of the *MAT* inversion (Figure 8).

### Segregation and DNA sequencing of the *MAT* inversion region in ordered tetrads

**Figure 9:**
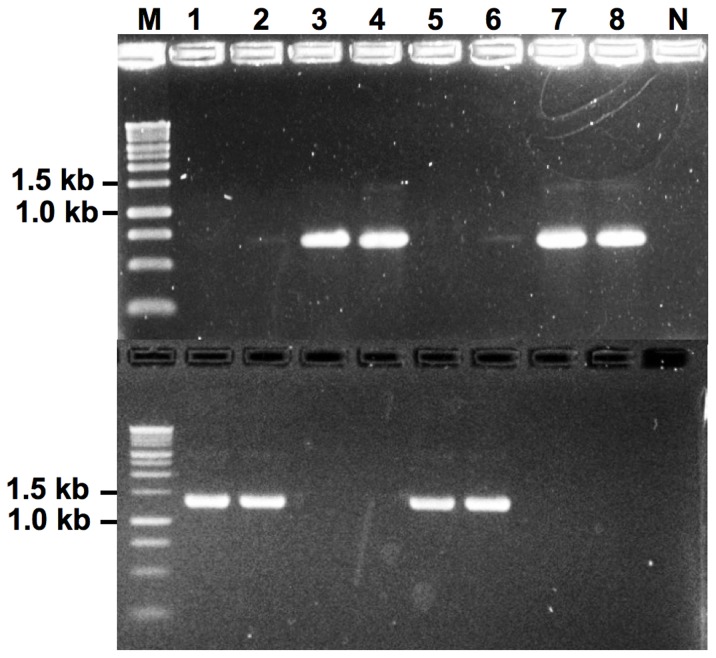
*MAT* locus distribution in the eight *Sclerotinia sclerotiorum* strains 1B331-1 – 1B331-8 representing an ordered tetrad. PCR gels with primers specific to Inv- (top) and Inv+ (bottom) *MAT* loci are shown (Figure S1). Lane numbers refer to *S. sclerotiorum* strain identifiers 1B331-1 – 1B331-8. The letters M and N indicate size markers and negative controls, respectively.

**Figure 10:**
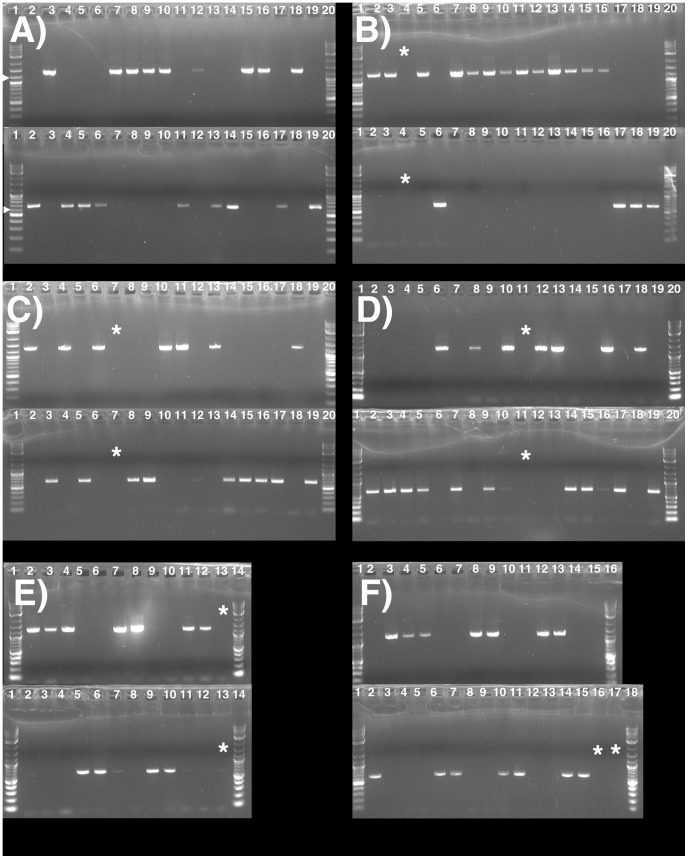
Orientation of *MAT* inversion region in *Sclerotinia sclerotiorum* isolates as evaluated by Inv+ and Inv- specific PCR reactions. Each isolate has one PCR band and is thus either Inv- or Inv+, weak bands in isolates with strong bands are false positives due to cross contamination. Isolates used are parental strains BS001, BS011, BS013, BS014, BS017 and BS028 (Table S1), and their progeny (Table S6), including the tetrads in Figure 10F. The top gel in each part figure shows the PCR bands obtained with primer pair Type-IIF / Type-IIR specific to Inv+, and the bottom gel the bands with primer pair MAT1-1-F / MAT1-1-R specific to Inv- (Figure S1). Lanes are numbered, lanes marked with asterisks contain negative controls, the first and last wells of each gel are DNA size standards, arrow heads in Figure 10A indicate positions of 1.2 kb and 0.6 kb bands, the remaining wells are as follows for *S. sclerotiorum* strains and negative controls in sequential order. 10A) BS011, BS011sa01, BS011sa02, BS011sa03, BS011sa04, BS011sa05, BS011sa06, BS011sa07, BS011sa08, BS011sa09, BS011sa10, BS011sa11, BS011sa12, BS011sa14, BS011sa15, BS011sa16, BS011sa17, BS011sa18. 10B) BS011sa19, BS011sa20, negative control, BS013, BS013sa01, BS013sa02, BS013sa04, BS013sa05, BS013sa06, BS013sa07, BS013sa08, BS013sa09, BS013sa10, BS013sa11, BS013sa12, BS013sa13, BS013sa14, BS013sa15. 10C) BS013sa16, BS013sa17, BS013sa18, BS013sa19, BS013sa20, negative control, BS017, BS017sa01, BS017sa02, BS017sa03, BS017sa04, BS017sa05, BS017sa06, BS017sa07, BS017sa08, BS017sa09, BS017sa10, BS017sa11. 10D) BS017sa12, BS017sa13, BS017sa14, BS017sa15, BS017sa16, BS017sa17, BS017sa18, BS017sa19, BS017sa20, negative control, BS028, BS028sa03, BS028sa04, BS028sa05, BS028sa06, BS028sa07, BS028sa08, BS028sa09. 10E) BS028sa10, BS028sa11, BS028sa12, BS028sa13, BS028sa14, BS028sa15, BS028sa16, BS028sa17, BS028sa18, BS028sa19, BS028sa20, negative control. 10F) BS001, BS014, 1B321-2, 1B321-4, 1B321-6, 1B321-8, 1B331-1, 1B331-2, 1B331-3, 1B331-4, 1B331-5, 1B331-6, 1B331-7, 1B331-8, negative control from upper gel, negative control from lower gel.

There was 1∶1 segregation of Inv- and Inv+ among the progeny of the two ordered tetrads examined. PCR screening for presence and absence of the inversion in the tetrads showed that among the eight *S. sclerotiorum* strains 1B331-1 – 1B331-8 of the complete tetrad, each sibling strain was PCR-positive for either the presence, or absence of the inversion (Figure 9). The *S. sclerotiorum* strains 1B331-1, 1B331-2, 1B331-5 and 1B331-6 originating from ascospores 1, 2, 5 and 6 numbered from top to bottom of the ascus, were Inv+, whereas *S. sclerotiorum* strains 1B331-3, 1B331-4, 1B331-7 and 1B331-8 corresponding to ascospores 3, 4, 7 and 8 were Inv- (Figure 9), indicative of crossing over and second division segregation [42], [43]. Similarly, among the four *S. sclerotiorum* strains 1B321-2, 1B321-4, 1B321-6 and 1B321-8 of the incomplete tetrad, *S. sclerotiorum* strains 1B321-2 and 1B321-4 were Inv+, whereas *S. sclerotiorum* strains 1B321-6 and 1B321-8 were Inv- (Figure 10F), in agreement with first division segregation [42].

Since the parent of the two tetrads was unknown, the two contending parental *S. sclerotiorum* strains were screened. *Sclerotinia sclerotiorum* strain BS001 was Inv-, and strain BS014 was Inv+ (Figure 10F).

The orientation of the *MAT* inversion regions and the flanking 250-bp motifs was confirmed by DNA sequencing in two isolates of the complete tetrad, *S. sclerotiorum* strain 1B331-1 that was Inv+, and strain 1B331-3 that was Inv-. The DNA sequences of the two *MAT* inversion regions and the flanking 250-bp motifs were identical, but the orientation of the *MAT* inversion regions differed between Inv- and Inv+ isolates as expected (Alignment S1).

### Proportions of Inv- and Inv+ isolates among random ascospore progeny

Random ascospore progeny consisted of equal proportions of Inv- and Inv+ isolates (Table 2). Progeny of four different parents were screened, including *S. sclerotiorum* strains BS011, BS013, BS017 and BS028 that were either Inv- or Inv+, and PCR analysis of 18 to 20 progeny for each parent showed that the ratio of Inv- to Inv+ isolates was 1∶1 for the progeny of individual parents, and for all progeny combined (Figure 10, Table S6).

**Table 2:** χ^2^ test results of observed versus expected Inv+ and Inv- frequencies among progeny from single apothecia of four parental strains, screening results are in Table S6.

Parent/Total	*Inv+*	*Inv-*	χ^2A^	χ^2A^	P value^B^
BS011	11	8	0.474	P = 0.49
BS013	13	6	2.579	P = 0.11
BS017	7	13	1.8	P = 0.18
BS028	10	8	0.222	P = 0.64
**Total**	**41**	**35**	**0.474**	**P = 0.49**

A Inv+ and Inv- frequencies were expected to be equal.

B None of the Inv+:Inv- ratios differed significantly from 1.

### Proportions of Inv- and Inv+ isolates among field populations

PCR screening for presence of the *MAT* inversion in *S. sclerotiorum* field populations in twelve different states showed that both types of *MAT* loci were present in California and Nebraska in equal proportions (Table 3). All isolates from Washington were Inv+, and for the remaining states only one or two isolates were screened (Table 3). Inv+ and Inv- isolates occurred on the same hosts that included lettuce, dry bean and canola, whereas in any given state, isolates from cauliflower, pepper, potato, soybean, sunflower and tobacco were either Inv- or Inv+. However, across states, soybean hosted both Inv+ and Inv- isolates (Table 3).

**Table 3:** Occurrence of *Sclerotinia sclerotiorum* Inv+ isolates in twelve states and on different hosts in the United States based on PCR screening of all isolates in Table S1.

Origin	Isolates screened	Inv+ isolates^A^	Hosts of Inv+ isolates	Hosts of Inv- isolates
California	220	99 (45%)^B^	Lettuce, dry bean, cauliflower	Lettuce, pepper
Georgia	2	1 (50%)	Canola	Canola
Illinois	1	0	-	Soybean
Kansas	1	0	-	Sunflower
Minnesota	1	0	-	Sunflower
Missouri	1	1	Soybean	-
Nebraska	26	16 (61%)^C^	Dry bean	Dry bean, soybean
North Dakota	1	1	Dry bean	-
Ohio	1	1	Soybean	
South Dakota	1	1	Soybean	
Wisconsin	1	0	-	Tobacco
Washington	15	15 (100%)	Potato	-

A Percentage is given when more than one isolate was screened.

B Inv- to Inv+ ratio was 1∶1 (χ^2^ test, P = 0.14).

C Inv- to Inv+ ratio was 1∶1 (χ^2^ test, P = 0.24).

### Comparison between inversion breakpoints of Inv- and Inv+ isolates

**Figure 11:**
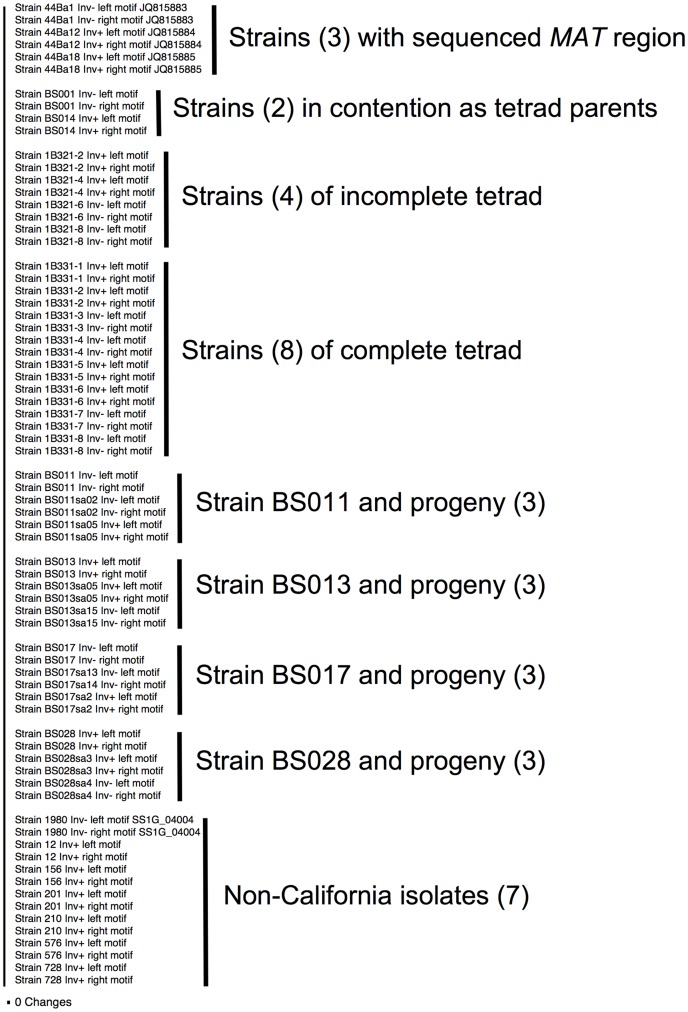
Phylogram inferred from 250-bp motifs of 36 *S. sclerotiorum* isolates. The two 250-bp motifs of each of the 36 isolates in Alignment S4 were aligned after the *MAT1-1-5* distal motifs were reverse complemented (Figure 3), resulting in a 72 taxa, 250 bp dataset (Alignment S5). Taxon names consist of strain identifier, inversion phenotype, and location of 250-bp motif with ‘left’ referring to *MAT1-1-5* proximal, ‘right’ to *MAT1-1-5* distal motifs (Figure 3). For sequences deposited or obtained from other sources, database accession numbers are also included. All isolates are listed in Table S1, except the isolates representing the random progeny of *S. sclerotiorum* strains BS011, BS013, BS017 and BS028 which are in Table S6. Groups of isolates are delimited on the right by vertical lines, numbers of isolates in each group are in parentheses. Included isolates were *S. sclerotiorum* strains 44Ba1, 44Ba12, 44Ba18 for which the entire *MAT* region was sequenced, *S. sclerotiorum* strains BS001 and BS014 that are in contention for parent of the two tetrads, *S. sclerotiorum* strains 1B321-2, 1B321-4, 1B321-6 and 1B321-8 of the incomplete tetrad, *S. sclerotiorum* strains 1B331-1 – 1B331-8 of the complete tetrad, *S. sclerotiorum* strains BS011, BS013, BS017 and BS028 with one Inv- and one Inv+ random progeny each, and the non-California isolates. There are no substitutions in the tree, illustrating that all 250-bp motifs have identical DNA sequences.

To confirm that the 250-bp motif was consistently present and intact on either side of the *MAT* inversion region, the inversion breakpoints were PCR amplified and sequenced in 32 different isolates of *S. sclerotiorum,* including 13 Inv- and 19 Inv+ isolates. We found that for each isolate, the inversion breakpoints contained the 250-bp motif as in the Inv- *S. sclerotiorum* strain 44Ba1 and the Inv+ strains 44Ba12 and 44Ba18 for which the entire *MAT* regions were sequenced (Alignment S4). All 250-bp motifs generated in this study and of *S. sclerotiorum* strain 1980 [17] were aligned after reverse complementing the *MAT1-1-5* distal 250-bp motifs, and the DNA sequences of all 250-bp motifs were identical (Figure 11, Alignment S5).

### Impact of the *MAT* inversion on self fertility

We investigated the relationship between *MAT* inversion and self fertility, and found that among the 36 *S. sclerotiorum* Inv+ isolates tested, 35 formed fully expanded apothecia which are the sexual fruiting bodies, and of the 21 Inv- isolates, 20 formed fully expanded apothecia (Table 4). Ascospore viability was not assessed in detail, but the ascospores of *S. sclerotiorum* strains BS001 and BS014 that are Inv- and Inv+, respectively, were viable [44], as were the ascospore progeny used for inversion screening (Table S6) and the 13 *S. sclerotiorum* strains used for assessment of mycelial compatibility groups (see below). In an apothecial stalk formation assay with another group of isolates, we found that of the 29 *S. sclerotiorum* Inv+ isolates, 18 produced apothecial stalks indicative of self-fertility (Table S7). All nine *S. sclerotiorum* Inv- isolates formed apothecial stalks (Table S7). However, the apothecial stalk assay may not necessarily reflect the ability to form mature apothecia [45].

**Table 4:** Apothecia production in 57 *Sclerotinia sclerotiorum* isolates from California.

Strain identifier	*MAT* inversion	Apothecium production, % A
BS001	Inv−	100
BS002	Inv−	95
BS003	Inv−	100
BS009	Inv+	100
BS010	Inv−	100
BS011	Inv−	100
BS012	Inv−	100
BS013	Inv+	95
BS014	Inv+	100
BS015	Inv+	95
BS016	Inv+	100
BS017	Inv−	75
BS018	Inv+	100
BS019	Inv+	100
BS020	Inv+	100
BS021	Inv+	100
BS022	Inv+	100
BS023	Inv+	95
BS024	Inv+	100
BS025	Inv+	100
BS026	Inv+	100
BS027	Inv+	100
BS028	Inv+	95
BS030	Inv+	100
BS031	Inv+	100
BS032	Inv−	100
BS033	Inv−	100
BS034	Inv−	100
BS035	Inv−	100
BS040	Inv+	100
BS041	Inv+	100
BS042	Inv+	0
BS047	Inv+	10
BS050	Inv+	100
BS051	Inv+	95
BS055	Inv+	100
BS057	Inv+	100
BS058	Inv+	30
BS061	Inv−	100
BS062	Inv+	95
BS063	Inv+	100
BS064	Inv+	100
BS065	Inv−	100
BS066	Inv−	100
BS067	Inv+	100
BS068	Inv+	100
BS070	Inv+	100
BS076	Inv−	100
BS079	Inv−	100
BS081	Inv−	95
BS082	Inv+	100
BS083	Inv+	100
BS084	Inv−	5
BS088	Inv−	0
BS089	Inv−	100
BS090	Inv+	45
BS096	Inv−	65

A Referring to the proportions of 20 sclerotia that gave rise to fully expanded apothecia over a four week period.

### Mycelial compatibility groups

All 13 *S. sclerotiorum* isolates investigated (BS001, BS002, BS003, BS011, BS013, BS014, BS017, BS028, BS047, BS058, BS071, BS095, BS096) (Table S1) were successfully paired with their progeny, and thus, mycelial compatibility groups were shared between parents and offspring in all instances. The *S. sclerotiorum* strains 1B331-1 – 1B331-8 representing the ordered tetrad were paired in all possible combinations among one another, and all pairings were compatible.

## Discussion

We investigated the *Sclerotinia sclerotiorum* mating type locus (*MAT*) in a collection of 283 isolates from lettuce in California and from other states and hosts, and found that *S. sclerotiorum* has two *MAT* alleles that differ in the orientation of a 3.6-kb region that is undergoing an inversion in every meiotic generation. The *MAT* inversion changes the orientation of two genes, truncates another gene and correlates with altered *MAT* gene expression. *MAT* inversions have also been reported in other ascomycetes including *Cochliobolus kusanoi* [24], *Peyronellaea* spp. [27] and *Stemphylium* spp. [25], but it is unknown whether in these fungi the inverted *MAT* regions are stable, or are inverted in every meiotic generation as in *S. sclerotiorum*.

### The *MAT* inversion region is present in half the progeny

**Figure 12:**
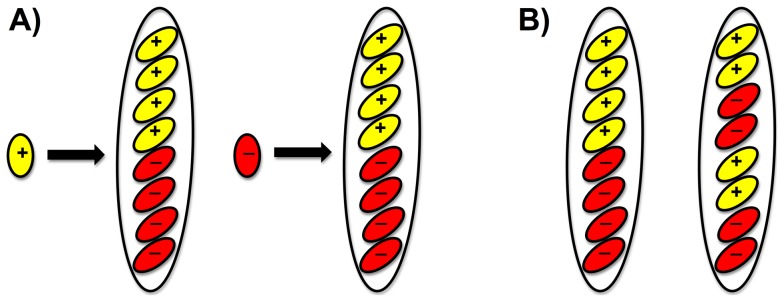
The *S. sclerotiorum MAT* inversion region changes orientation in every meiotic generation and segregates at the first or second division of meiosis. Small ovals are ascospores, large ovals are asci, yellow ascospores are Inv+, red ascospores are Inv-. Single ascospores represent parental isolates, asci containing eight ascospores represent progeny. 12A) Regardless of whether the parent is Inv- or Inv+, 50% of the progeny is Inv+, suggesting a highly regulated process during the sexual cycle ensures the 1∶1 ratio. The transition of Inv- to Inv+ involves a change in orientation of the *MAT* inversion region. 12B) Meiotic segregation pattern of Inv- and Inv+ among ascospores in *S. sclerotiorum*. The pattern that is characteristic for first division segregation in *Neurospora crassa* is shown on the left, the pattern for second division segregation following recombination is shown on the right [42]. Figure 12 is based on PCR screens for absence and presence of the *MAT* inversion region in tetrad and random progeny and their parents with DNA from the mycelial phase (Figures 9, 10), DNA sequencing of inversion breakpoints (Figure 11) and the entire inversion regions and flanks in *S. sclerotiorum* strains 1B331-1 and 1B331-3 of the complete ordered tetrad (Alignment S1), and Southern blotting illustrating that *S. sclerotiorum MAT* is single copy (Figure 8).

The process associated with the change in orientation of the 3.6-kb *MAT* inversion region in *S. sclerotiorum MAT* may be highly regulated, because it results in a 1∶1 ratio of ascospores that have the inversion and that are referred to as Inv+, to ascospores without the inversion designated Inv-. The 1∶1 ratio was observed among random progeny and in ordered tetrads. Half of the 18 to 20 randomly selected ascospores of each of the four apothecia examined, were Inv+ and half were Inv-, regardless of whether the parental isolate was Inv+ or Inv- (Figure 12A). And the ratio of Inv+ to Inv- ascospores in a single ascus was 1∶1. The two asci examined displayed inversion distribution patterns among the sibling ascospores indicative of segregation and recombination (Figure 12B).

The 1∶1 ratio of Inv+ to Inv- isolates was also found at the population level in California and Nebraska suggesting that the transition between Inv- and Inv+ that we documented in the laboratory, may also commonly occur in nature (Table 3). Inv+ isolates were widespread, and in addition to California and Nebraska, were also found in Georgia, Missouri, North Dakota, Ohio, South Dakota and Washington State, but with the exception of Washington where all 15 isolates tested were Inv+, no more than two isolates were included for each of these states (Table 3). Based on our dataset, both Inv- and Inv+ isolates were obtained from lettuce, canola and dry bean from California, Georgia and Nebraska, respectively. For all other states only single isolates were screened (Table S1, Table 3), with the exception of potato in Washington where all 15 isolates were Inv+ (Table 3).

### *MAT* inversion does not occur during mycelial growth and sclerotia formation

We did not investigate in detail when during the *S. sclerotiorum* life cycle and under which conditions the *MAT* inversion occurs, but based on PCR results it appears that the rearrangement of the *MAT* inversion region does not occur during vegetative hyphal growth or during sclerotium formation. This is because the mycelia used for DNA extraction were grown from sclerotia that were generated in the laboratory, and all strains were either Inv- or Inv+ based on PCR assays, and none were both (Figure 10). Also, the inversion phenotype of *S. sclerotiorum* strain 1B331-8 did not change following subculturing (data not shown). This is dissimilar to human blood cells where DNA regions flanked by inverted repeats similarly to the *MAT* inversion region, undergo recurrent inversions that result in cell populations that are mixed with regard to the orientations of inversion regions [46]. However, we expect a mixed population of nuclei within the same structure following rearrangement of the *MAT* inversion region, as *S. sclerotiorum* asci harbor both Inv- and Inv+ ascospores (Figures 8, 10, 12).

Under appropriate conditions, sclerotia in *S. sclerotiorum* give rise to apothecia that are sexual fruiting bodies where meiosis occurs within the asci, and since we did not detect rearrangement of the *MAT* inversion region during vegetative growth, inversion appears to occur during the sexual cycle. The arrangement of Inv- and Inv+ ascospores inside the asci is consistent with first and second division segregation during meiosis (Figure 12B), which suggests that Inv- and Inv+ nuclei were paired up prior to meiosis, and thus, the inversion precedes meiosis. Furthermore, pairing of Inv- and Inv+ nuclei may require nuclear recognition, a process that in heterothallic ascomycetes involves *MAT* and the production of nucleus-specific hormones and hormone receptors [32], [33], [34], [35]. *Sclerotinia sclerotiorum* is homothallic, but Inv+ and Inv- *MAT* regions differ in gene expression (Figure 6) and may be functionally equivalent to *MAT1-1* and *MAT1-2* of heterothallics, also because Inv+ *MAT* appears to lack a functional alpha1 box (Figure 7). Thus, we hypothesize that rearrangement of the *MAT* inversion region occurs before meiosis but following sclerotium formation, and is involved in recognition between Inv- and Inv+ nuclei.

We do not know what triggers the *MAT* inversion, but in human blood cells, the recurrent inversion events are caused by non-allelic homologous recombination [46], a process that is initiated through DNA double strand breaks [47]. Non-allelic homologous recombination that results in genome rearrangements is also known from *Saccharomyces cerevisiae* [48].

### *MAT* inversion is facilitated by two 250-bp motifs

The trigger for the *MAT* inversion is unknown. But the inversion event is most likely caused by non-allelic homologous recombination [46], [47] between the 250-bp motifs that flank the 3.6-kb *MAT* inversion region. The *MAT1-1-5* distal 250-bp motif is inverted with respect to the *MAT1-1-5* proximal 250-bp motif, and juxtaposition of the two motifs and recombination results in a 3.6-kb inversion without altering the DNA sequence composition of the 250-bp motifs (Figure 3). Since one 250-bp motif is located near the center of *MAT1-1-1,* and the other 250-bp motif overlapped with the 3’-end of *MAT1-2-1,* only *MAT1-1-1* is truncated by the inversion (Figure 3; Alignment S1). In all 36 isolates examined, the two 250-bp motifs had identical DNA sequence composition (Figure 11). We sequenced across the two 250-bp motifs and the 3.6-kb *MAT* inversion region in *S. sclerotiorum* strains 1B331-1 (Inv+) and 1B331-3 (Inv-), two progeny of the ordered tetrad, and did not find any differences between the two isolates apart from the orientation of the *MAT* inversion region (Alignment S1).

### *MAT* locus is single copy

There is just one *MAT* locus in *S. sclerotiorum*, based on the analysis of strain 1980 [17]. And the results from Southern blotting with a *MAT1-2-1* probe (Figure 8) were consistent with the presence of either Inv+ or Inv- *MAT* as documented by PCR screening (Figure 10). Thus, the transition between Inv- and Inv+ isolates is not due to the exchange of the *MAT* inversion region between two *MAT* loci, and thus differs from mating type switching in baker’s yeast, *Saccharomyces cerevisiae* [49].

### Evolutionary origin of the *MAT* inversion region

*Sclerotinia sclerotiorum* is closely related to *B. cinerea* [50], [51], but the two fungi differ in mating system and *MAT* gene arrangement. Whereas *B. cinerea* is heterothallic and each individual has a *MAT1-1* or a *MAT1-2* idiomorph at *MAT* [17], *S. sclerotiorum* is homothallic and the two *MAT* idiomorphs are fused, similarly to *Cochliobolus* [24], *Crivellia* [26], *Didymella* and *Peyronellaea* [27], *Gibberella* [52] and *Stemphylium* [25]. Homothallic species are generally thought to have evolved from heterothallic ancestors [24], [25], [26], [27], and a similar scenario is possible for *S. sclerotiorum* (Figure 4C).

The *S. sclerotiorum MAT* arrangement resembles most closely the *MAT* arrangement of homothallic *Stemphylium* species where the *MAT1-1* idiomorph is inverted with respect to heterothallic relatives, and is fused to *MAT1-2* [25]. The fused *MAT* arrangement in *Stemphylium* evolved following the inversion of *MAT1-1* that created a short stretch of DNA sequence identity between idiomorphs, facilitating a crossing over that resulted in the *MAT1-1 – MAT1-2* fusion [25]. Similarly to *Stemphylium*, the orientation of *MAT1-2* differs between *S. sclerotiorum* and *B. cinerea* [17]. To investigate whether in an ancestor of *S. sclerotiorum* the *MAT1-2* inversion could have facilitated crossing over resulting in *MAT* fusion, we looked for a crossover site and aligned the *MAT1-1 – MAT1-2* fusion junction in *S. sclerotiorum* Inv- isolates with homologous regions of *B. cinerea*, a proxy for the unknown ancestor. We found that the fusion of *MAT1-1* and *MAT1-2* most likely occurred in a region of 10 bp encompassing nucleotides 239 to 248 downstream of *S. sclerotiorum MAT1-1-1*. This is because DNA sequence homology between *B. cinerea* and *S. sclerotiorum MAT1-1* idiomorphs extends 248 nucleotides downstream of *S. sclerotiorum MAT1-1-1*, and among the ten nucleotides in positions 239 to 248 downstream of *S. sclerotiorum MAT1-1-1,* five were identical between *B. cinerea MAT1-1*, *S. sclerotiorum MAT1-1* and *B. cinerea MAT1-2* (Figure 5; Alignment S3). The relatively high DNA sequence identity between *B. cinerea MAT1-1* and *MAT1-2* idiomorphs at the *S. sclerotiorum MAT1-1 – MAT1-2* fusion junction is consistent with the presence of an ancient crossover site. Unlike *MAT1-1*, the *B. cinerea* and *S. sclerotiorum MAT1-2* regions were generally too divergent to be aligned with confidence outside of the presumptive crossover site (Alignment S3).

In other homothallic ascomycetes, *MAT1-1 – MAT1-2* fusion junctions with similarities to homologous regions of related heterothallics are also known. Between the homothallic *Cochliobolus luttrellii* and its heterothallic relative, the fusion junction consisted of seven consecutive nucleotides shared between the two species [24]. Similarly, in *Crivellia* there were four shared consecutive nucleotides [26], in *Didymella* three [27] and in *Stemphylium* four [25].

An alternative explanation for the evolution of the *S. sclerotiorum* and *B. cinerea MAT* loci has been proposed. It was suggested that the fused idiomorphs of *S. sclerotiorum* were ancestral, and the separate idiomorphs of *B. cinerea* were derived [17]. This evolutionary scenario is less likely, since it requires at least three different steps to occur in an ancestor of *B. cinerea*, including a loss of *MAT1-1*, a loss of *MAT1-2* and an inversion of *MAT1-2*. Our scenario is more parsimonious since it requires only two steps which are inversion of *MAT1-2*, and fusion of *MAT1-2* to *MAT1-1*. The presence of partial *MAT1-1-1* and *MAT1-2-1* genes in *B. cinerea MAT*, proposed remnants of incompletely deleted *MAT1-1-1* and *MAT1-2-1* [17], likely resulted by crossing over between *B. cinerea MAT1-1* and *MAT1-2* after evolutionary separation from the *S. sclerotiorum* lineage.

A further difference between *B. cinerea* and *S. sclerotiorum MAT* is the presence of the 250-bp motifs delimiting the 3.6-kb *MAT* inversion region in *S. sclerotiorum*. Since a 250-bp motif is absent in *B. cinerea MAT1-2-1*, but present in *MAT1-1-1* of both *B. cinerea* and *S. homoeocarpa* (Table S5; Alignment S2), the 250-bp motif is most likely a partial *MAT1-1-1* that was integrated into *MAT1-2-1* in an ancestor of *S. sclerotiorum*, possibly through a double crossing over (Figure 4A). However, no potential crossover sites were found when comparing the flanks of the 250-bp motif in *S. sclerotiorum* to homologous regions of *B. cinerea MAT1-1* and *MAT1-2*. Transposons are known to mediate inversions, for instance in bacteria [53] and in *Drosophila* [54], [55], but the 250-bp motif did not contain any transposon sequences and lacked repeats.

### *MAT* inversion correlates with *MAT* gene expression

Gene expression was examined in the eight *S. sclerotiorum* strains 1B331-1 – 1B331-8 of the complete tetrad, and differences in expression of *MAT1-1-1* and *MAT1-2-1* were found between Inv- and Inv+ isolates (Figures 6, 7). In each of the four Inv- isolates, there were three *MAT1-2-1* transcript variants, two of which did not encode any known proteins. The third variant encoded an HMG protein, and was the only *MAT1-2-1* transcript present in the *S. sclerotiorum* strain 1980 transcriptome from the recent *S. sclerotiorum* genome sequencing project [17]. Inv- isolates contained a single *MAT1-1-1* transcript variant encoding an alpha1 protein (Figures 6, 7), but only the 3’-end of the transcript was found in the *S. sclerotiorum* strain 1980 transcriptome. The four Inv+ isolates had only one of the three *MAT1-2-1* transcript variants of Inv- isolates, and a different *MAT1-1-1* variant. The Inv+ *MAT1-2-1* variant encoded an HMG protein, and the Inv+ *MAT1-1-1* variant encoded a truncated alpha1 protein, lacking all but the first N-terminal 15 alpha1 box residues of the 197 residues that are present in Inv- isolates. As expected, no full-length match to the Inv+ *MAT1-1-1* variant was found in the *S. sclerotiorum* strain 1980 transcriptome that was Inv-. Reasons for the absence of *MAT1-2-1* transcript variants 1 and 3 (Figure 7) from the *S. sclerotiorum* strain 1980 transcriptome may include incomplete transcriptome sequencing coverage, or alteration in *MAT* gene expression patterns due to differences in growth conditions. Multiple transcripts for *MAT1-1-1* and *MAT1-2-1* were also present in *Cochliobolus heterostrophus* [56], and alteration of gene expression upon gene inversion was also documented in *Drosophila* [54], [55].

Based on our *MAT* gene expression data (Figure 7), the severely truncated MAT1-1-1 of *S. sclerotiorum* Inv+ isolates is expected to be non-functional, and thus, Inv+ isolates are expected to be functionally equivalent to heterothallic *MAT1-2* isolates that generally are unable to self. However, we found that *S. sclerotiorum* Inv+ isolates were able to self, indicating that the truncated MAT1-1-1 may be functional, that a complete MAT1-1-1 may be expressed in a different part of the life cycle, or that Inv+ isolates are able to self without a functional MAT1-1-1. It is known that co-existence of *MAT1-1-1* and *MAT1-2-1* within one genome is not always required for selfing, as certain *Stemphylium* species and *Neurospora africana* are homothallic, but contain only a single *MAT* idiomorph [25], [57].

Altered *MAT* gene expression did not have any influence on mycelial compatibility groups, as for all 13 parental strains tested, parent and progeny were of the same mycelial compatibility group, as were the *S. sclerotiorum* strains 1B331-1 – 1B331-8 representing the ordered tetrad.

### *Sclerotinia sclerotiorum* has two MAT alleles

The different versions of the *MAT* locus within one species are typically referred to as idiomorphs, due to their general lack of DNA sequence homology. The case of *S. sclerotiorum* is different, the Inv- and Inv+ versions of *MAT* differ mainly by an inversion (Figure 1), and it is readily apparent that they are ‘related in structure and descent’ unlike idiomorphs [14]. We thus propose to refer to the two versions of *MAT* in *S. sclerotiorum* as alleles. This may not be entirely accurate since alleles typically refer to different versions of a single gene and not groups of genes, but the term allele instead of idiomorph was previously used to refer to different versions of the mating type locus of *Neurospora crassa* [14].

### Presence of a *MAT* inversion region could explain mating type switching in filamentous fungi

In several species of filamentous ascomycetes, only half the progeny derived from a selfing parent is able to self, and the other half is self-sterile but is able to outcross to self-fertile strains. Thus, in these filamentous ascomycetes, there is a transition in life style from homothallism to heterothallism that occurs within a single generation, a process known as mating type switching [58] that differs from mating type switching in baker’s yeast, *Saccharomyces cerevisiae* [28]. Examples of filamentous ascomycetes where mating type switching has been demonstrated include *Chromocrea spinulosa* [21], *S. trifoliorum* [22] and certain *Ceratocystis* species [23].

In progeny of *Ch. spinulosa* and *S. trifoliorum*, mating type correlates with ascospore size dimorphism. Heterothallic progeny show a decrease in ascospore size with respect to homothallic progeny [21], [40]. Similarly, heterothallic strains of *Ce. fimbriata* grow more slowly than homothallic strains [23]. Thus, altered ascospore size or growth rate may represent pleiotropic effects of *MAT*, similarly to *Coniochaeta tetraspora* where half of the eight ascospores in each ascus are aborted [59].

Details on mating type switching in filamentous ascomycetes are unknown, but the involvement of a chromosomal inversion has previously been proposed [58]. A *MAT* inversion region similar to the one in *S. sclerotiorum* could account for mating type switching and the other phenotypic changes observed in half the progeny of *Ch. spinulosa* [21], *S. trifoliorum* [22], [40] and in *Ce. fimbriata* [23]. Upon inversion, *MAT* genes that are situated on an inversion breakpoint are truncated, and *MAT* genes that are near an inversion breakpoint may undergo a change in gene expression, as observed in *Drosophila* [55]. Since *MAT* genes are pleiotropic and encode transcription factors that regulate more than 150 target genes, the majority of which have unknown functions [31], altered expression of just one *MAT* gene may affect multiple phenotypic traits, including mating system, ascospore size and viability, and growth rate. Thus, a *MAT* inversion region has the potential to act as a switch that regulates different sets of genes depending on its orientation. In the *S. sclerotiorum MAT* inversion region, the inversion is reversible, as the progeny of both Inv- and Inv+ parents is 50% Inv+. But in other ascomycetes, a one-time transition from Inv- to Inv+ in every meiotic generation would be sufficient to account for the mating type switching and the other phenotypic changes that have been documented. The inversion process may be mediated by inverted, non-repetitive DNA as is present at the inversion breakpoints of the *S. sclerotiorum MAT* inversion region. Alternatively, transposons are known to mediate inversions, including in bacteria [53] and in *Drosophila* [54], [55].

Molecular data on mating type switching does not contradict involvement of an inversion. In *Ceratocystis* spp., Witthuhn et al. [60] used HMG box specific PCR primers and found that PCR amplification failed in heterothallic isolates. This would be expected if the HMG box comprised an inversion breakpoint. In *Ch. spinulosa*, homothallic strains have a *MAT1-2-1* adjacent to a *MAT1-1* idiomorph, the *MAT1-2-1* is missing in that position in heterothallic isolates [61]. There is evidence for inversion breakpoints in homothallic *Ch. spinulosa* isolates in the form of repeats situated on either side of *MAT1-2-1*. One of the repeats is present upstream of *MAT1-2-1*, the other downstream of *MAT1-2-1*, inside *MAT1-1*. Since the distance between upstream repeat and *MAT1-2-1* is greater than the distance between downstream repeat and *MAT1-2-1*, inversion of the region between the repeats would increase the distance between *MAT1-2-1* and *MAT1-1*. This could explain why *MAT1-2-1* was not detected near *MAT1-1* in heterothallic isolates. However, more research on the nature of mating type switching in *Ch. spinulosa* and other species should include comparisons of genomes and gene transcription profiles between parents and offspring.

### Further research

Given the importance to agriculture, it is surprising how little is known about sexual processes in *S. sclerotiorum*. Future research should focus on elucidating when and how the inversion occurs, whether there are differences in target gene expression between Inv- and Inv+ strains, and whether the inversion phenotype plays a role in fertilization and outcrossing.

## Materials and Methods

### Fungal strains and culture conditions

This study was based on 283 *Sclerotinia sclerotiorum* isolates of which 214 were from lettuce in California. The remaining 69 isolates were from nine other substrates (canola, cauliflower, dry bean, field soil, pepper, potato, soybean, sunflower, and tobacco) from twelve states (California, Georgia, Illinois, Kansas, Minnesota, Missouri, North Dakota, Nebraska, Ohio, South Dakota, Washington and Wisconsin), or were part of ordered tetrads obtained under laboratory conditions (Table S1). Fungi were grown on potato dextrose agar medium (PDA) (Sigma-Aldrich, St. Louis, MO) and potato dextrose broth (PDB) (Sigma-Aldrich, St. Louis, MO) at room temperature. California isolates were derived from sclerotia collected from infected plants. One sclerotium per plant was surface sterilized in 10% bleach for 3 min, plated on PDA, and hyphal tips were transferred to fresh PDA plates to obtain pure cultures. Stocks were maintained as sclerotia at 4°C.

Additional strains used in this study were progeny obtained as random ascospore isolates from strains listed in Table S1. Ascospore suspensions were prepared as described by Wu et al. [44], diluted, spread out on PDA, and single germinated ascospores were transferred to new PDA plates by excising the agar block containing the ascospore using a forceps. Progeny are not included in Table S1, but parental strains are mentioned in the text.

### DNA sequences from GenBank and the Broad Institute

DNA sequences of the *S. sclerotiorum* strain 1980 *MAT* region including flanking genes were retrieved from the Broad Institute website (http://www.broadinstitute.org, accessed in September 2011) (*APN2*: SS1G_04002; *MAT1-1-5*: SS1G_04003; *MAT1-1-1*: SS1G_04004; *MAT1-2-4*: SS1G_04005; *MAT1-2-1*: SS1G_04006; *SLA1*: SS1G_04007) and the coding sequences for *APN2*, the *MAT* genes and *SLA2* were from GenBank (XM_001594145 - 50). *Botrytis cinerea* sequences were from GenBank for the *MAT1-1* region (AAID01003685), and the *MAT1-2* gene and coding sequences (FQ790352), from the Broad Institute website (http://www.broadinstitute.org, accessed in September 2011) for the *MAT1-1* gene sequences (*MAT1-1-5*: BC1G_15147; *MAT1-1-1*: BC1G_15148), and from GenBank for the *MAT1-1* coding sequences (XM_001546387 - 88).

### Nucleic acid extraction

*Sclerotinia sclerotiorum* mycelia were grown at room temperature in 25 ml PDB on a lab bench, inoculated with three to four day old PDA cultures, and harvested after four days. For PCR, DNA was extracted by FastDNA extraction kit (MP Biomedicals, Solon, OH). For Southern blotting, a standard phenol-chloroform protocol was used [62]. Total RNA was extracted using TRIZOL (Invitrogen Life Technologies, Carlsbad, CA) following the manufacturer’s protocol. The integrity of the RNA was verified by agarose gel electrophoresis. Nucleic acids were quantified using a NanoDrop system (NanoDrop Technologies, Wilmington, DE) and the DNA concentration was adjusted to 2-10 ng/µl in PCR grade water for PCR amplification and to 5–8 µg for Southern blotting.

### Cloning of *MAT* loci, phylogenetic analyses and identification of repeated motifs

Complete *MAT* locus coverage of *S. sclerotiorum* strains 44Ba1, 44Ba12 and 44Ba18 was obtained by PCR using primers designed based on the *S. sclerotiorum* strain 1980 *MAT* locus sequences [17]. Three overlapping PCR reactions targeted the *MAT* locus and parts of the flanking *APN2* and *SLA2*, using primer pairs MAT_1069F / MAT_6555R, MAT_6042F / MAT_11930R and MAT_11655F / MAT_14587R (Table S8). PCRs were performed in 50 µl reactions consisting of 25 µl GoTaq Green Master Mix (Promega Corp., Madison, WI), 1.5 µl of each primer (10 pmol/µl), 5 µl of genomic DNA (2 ng/µl) and 17 µl of PCR grade water. PCR amplifications were carried out on a Bio-Rad DNA Engine thermocycler (Bio-Rad Laboratories, Hercules, CA) with the following conditions: Initial denaturation at 95°C for 10 min, followed by 35 cycles of 95°C for 20 sec, 60°C for 30 sec, and 60°C for 5 min, with a 10 min final extension at 72°C [63]. Aliquots of PCR products (6µl) were separated on a 1% agarose gel by electrophoresis, gels were stained with ethidium bromide and visualized under a UV trans-illuminator (Ultra-Violet Products Ltd, Cambridge, UK). PCR products were sequenced with the PCR primers and additional internal primers were designed to generate complete sequencing coverage in both directions for all three isolates. Primer sequences are given in Tables S8, S9, and S10. DNA sequencing was performed at the UC Davis DNA Sequencing Facility, using ABI BigDye Terminator v3.1 Cycle Sequencing chemistry on an ABI 3730 Capillary Electrophoresis Genetic Analyzer (Applied Biosystems, Foster City, CA, USA). Sequences were assembled by Geneious v4.8.5 [64]. The *S. sclerotiorum* strains 44Ba1, 44Ba12 and 44Ba18 *MAT* locus sequences were deposited in GenBank (JQ815883, JQ815884, and JQ815885, respectively).

Phylogenetic analyses of the four *MAT* genes were inferred using PAUP v.4.0b 10 [65] using parsimony with default settings including treating gaps as missing data. Alignments were generated in Geneious v4.8.5 [64] invoking CLUSTAL X version 2.1 [66], [67], and are available as Alignment S6, Alignment S7, Alignment S8 and Alignment S9. All four alignments contained five taxa, including *B. cinerea* as outgroup, as well as *S. sclerotiorum* strains 1980, 44Ba1, 44Ba12 and 44Ba18.

The 250-bp motif sequence was analyzed for the presence of repeats using REPFIND [68], available at http://zlab.bu.edu/repfind/index.shtml.

### PCR screening for *MAT* inversion and sequencing of inversion breakpoints

All *S. sclerotiorum* isolates in Table S1 and the progeny of *S. sclerotiorum* strains BS011, BS013, BS017 and BS028 listed in Table S6 were screened for the presence and absence of the *MAT* inversion using two sets of PCR primers. The Inv+ specific primer pair Type-IIF / Type-IIR (Table S11) targeted a 1306 bp fragment spanning the *MAT1-1-5* proximal inversion breakpoint (Figure S1), and the primer pair MAT1-1-F / MAT1-1-R targeted a 671 bp fragment of the intact *MAT1-1-1* of Inv- isolates [69]. Primer binding sites are illustrated in Figure S1. PCR conditions were as described for *MAT* locus cloning, except that the extension temperature and time were 72°C and 60 sec, respectively. PCR reactions were performed on a Bio-Rad DNA Engine thermocycler (Bio-Rad Laboratories, Hercules, CA). Aliquots of PCR products (6 µl) were separated on a 1% agarose gel by electrophoresis, stained with ethidium bromide and visualized under UV trans-illuminator (Ultra-Violet Products Ltd, Cambridge, UK). Selected PCR products were sequenced to confirm target-specific amplification.

Both inversion breakpoints were PCR amplified and sequenced for the 18 *S. sclerotiorum* strains BS001, BS011, BS013, BS014, BS017, BS028, 1B321-2, 1B321-4, 1B321-6, 1B321-8, and 1B331-1 – 1B331-8 from California (Table S1), and from one Inv- and one Inv+ randomly selected progeny of strains BS011, BS013, BS017 and BS028, respectively (Table S6). Also sequenced were the inversion breakpoints of the Inv+ *S. sclerotiorum* strains 12, 156, 201, 210, 576, 728 that originated from outside California (Table S1). Primer pairs used were SS1f / SS2f2 and SS1r2 / SS2r in Inv- isolates for the *MAT1-1-5* proximal and distal inversion breakpoints, respectively, targeting 458 and 677 bp, respectively. Primer pairs SS1f / SS1r2 and SS2f2 / SS2r were used for Inv+ isolates for the *MAT1-1-5* proximal and distal inversion breakpoints, respectively, targeting 554 and 564 bp, respectively (Figure S1, Table S11). The following conditions were used: Initial denaturation at 94°C for 2 min, followed by 32 cycles of 94°C for 10 sec, 55°C for 20 sec, and 72°C for 1 min, with a 7 min final extension at 72°C. DNA sequencing was performed as described above.

The *MAT* inversion region was PCR amplified and sequenced for two of the progeny of the complete tetrad, *S. sclerotiorum* strain 1B331-1 (Inv+) and 1B331-3 (Inv-). PCR primers used were SS1f / SS2r, targeting 4.4. kb including the *MAT* inversion region and the 250-bp motifs, using GoTaq Colorless Master Mix (Promega Corp., Madison, WI, USA) and the following PCR conditions based on Garza et al. [63]: Initial denaturation at 94°C for 3 min, followed by 35 cycles of 94°C for 10 sec, 60°C for 20 sec, and 60°C for 6 min, with a 7 min final extension at 60°C. The resulting PCR bands were cut out from gel, melted in 50 µl PCR quality water, diluted 10 and 100 times, and used as templates in PCR reactions with three overlapping primer pairs, SS1f / SSr5, SSf2 / SSr2, SSr4 / SS2r, for *S. sclerotiorum* strain 1B331-1, and primer pairs SS1f / SSr1, SSf2 / SSr2, SSf3 / SS2r, for *S. sclerotiorum* strain 1B331-3. The PCR primers, as well as primer SSr7 for *S. sclerotiorum* strain 1B331-1, and primers SSf5 and SSf6 for *S. sclerotiorum* strain 1B331-3 were used for DNA sequencing as described above (Table S11).

### *MAT* gene expression analysis

The expression of all *MAT* genes was assessed using primer pairs specific to each gene (Figure 6), in *S. sclerotiorum* strains 1B331-1, 1B331-2, 1B331-5 and 1B331-6 that are Inv+, and strains 1B331-3, 1B331-4, 1B331-7 and 1B331-8 that are Inv-. The primer pairs for each gene and approximate expected amplicon sizes were as follows. *MAT1-1-5*: MAT5F / MAT5R (350 bp); *MAT1-1-1*: MAT1F / MAT1R (250 bp); *MAT1-2-4*: MAT4F / MAT4R (310 bp); *MAT1-2-1*: MAT2F / MAT2R (1180 bp); *MAT1-1-1* 3’-end fragment: FusionF / FusionR (640 bp), and for the *actin* control (250 bp): F / R (Table S12). First-strand cDNA synthesis was done with DNAse treated total RNA using Clontech SMARTer cDNA synthesis kit following the manufacturer’s instructions (Clontech Laboratories, Inc., CA). Double-stranded cDNA for all *MAT* genes was synthesized using the gene-specific primer pairs listed in Table S12.

When more than one transcript was obtained, PCR products were cloned into pCR2.1-TOPO following the manufacturer’s protocol (Invitrogen Life Technologies, Carlsbad, CA) and sequenced using both vector (M13F / M13R) and gene specific primers (Table S5). Exon-intron boundaries were determined by comparison of transcript and gene sequences using CLUSTALW V2.1 [66].

### Southern blotting of *MAT* region

To assess the numbers of *MAT* loci in *S. sclerotiorum* genomes, Southern blotting was performed for *S. sclerotiorum* isolates derived from the ordered tetrad. These were the *S. sclerotiorum* Inv+ strains 1B331-1, 1B331-2, 1B331-5 and 1B331-6, and the *S. sclerotiorum* Inv- strains 1B331-3, 1B331-4, 1B331-7 and 1B331-8. Genomic DNA (5 to 8 µg) was digested with *Bsa*HI that had restriction sites upstream, downstream and on the *MAT* inversion (Figure 8). Digests were run on 0.8% agarose gel and transferred to nylon membrane (Roche, Basel, Switzerland) following Selden [70]. A Southern probe specific to *MAT1-2-1* (Figure 8) was generated by PCR using primers MAT1-2-F / MAT1-2-R [69]. The probe was labeled with digoxigenin (Roche, Basel, Switzerland) according to the manufacturer’s instructions, and hybridization and detection was performed according to the manufacturer instructions (Roche, Basel, Switzerland). X-ray film (Kodak, Rochester, NY) was used and developed following a 30 min exposure. The probe was expected to hybridize to 1.16 and 1.5 kb fragments in Inv- and Inv+ isolates, respectively.

### Generation of the sexual state in culture

Apothecium formation was evaluated following the method of Wu et al. [44]. Two different groups of isolates were used. The production of fully expanded apothecia was assessed for a group of 57 isolates based on 20 sclerotia per strain (Table 4). The formation of apothecial stalks, an approximate measure of apothecia formation, was assessed for another group of 38 isolates (Table S7), and repeated once for isolates that failed to produce apothecial stalks in the first repetition.

### Isolation of ordered tetrads

Ordered tetrads were obtained by culturing all ascospores of a single ascus. Asci were derived from a fully expanded apothecium grown in culture [44] and were released by placing apothecia in 1.5 ml centrifuge tubes, adding sterile water and gentle macerating by forceps. Random asci containing eight mature ascospores were squeezed from the distal end to release the ascospores using a scalpel, and ascospores were transferred in sequence to individual PDA plates and stored at 4°C. Two tetrads were obtained, a complete tetrad was represented by the eight *S. sclerotiorum* strains 1B331 – 1B331-8, another tetrad was incomplete and consisted of the four *S. sclerotiorum* strains 1B321-2, 1B321-4, 1B321-6 and 1B321-8. The numbers following the dashes reflect the ascospore positions inside the ascus. For instance, *S. sclerotiorum* strain 1B331-1 was derived from the ascospore closest to the tip of the ascus. Tetrads were isolated from two different parental isolates, *S. sclerotiorum* strains BS001 and BS014. Our lab notes were inconclusive as to which of the two strains was the parent of the tetrads used in this study. Strains BS001 and BS014 were identical at the variable *MAT* regions that were sequenced and compared (data not shown).

### Mycelial compatibility group assessment

Mycelial compatibility groups were assessed for 13 *S. sclerotiorum* isolates (BS001, BS002, BS003, BS011, BS013, BS014, BS017, BS028, BS047, BS058, BS071, BS095, BS096) (Table S1) and their progeny, at least 20 progeny were tested for each parent. Progeny were paired with their parent as described by Wu and Subbarao [3] following the methodology of Schafer and Kohn [71]. *Sclerotinia sclerotiorum* strains 1B331-1 – 1B331-8 representing the complete ordered tetrad were paired in all possible combinations among one another.

## Supporting Information

Figure S1 **Positions of primers used for** ***MAT*** **inversion screening (MAT1-1F / MAT1-1R, Type-IIF / Type-IIR), and PCR amplification and sequencing of the inversion breakpoints in** ***S. sclerotiorum*** **isolates S1A) without (Inv-) and, S1B) with inversion (Inv+).** Genes are boxes, white and dotted boxes correspond to alpha1 and HMG domains, respectively, directions of transcription are indicated by arrows, gene names are inside or by the boxes. Dashed box and arrow represent *MAT1-1-1* 3’-end fragment lacking an in frame start codon. Primer sites are indicated by half arrows. Diagrams are to scale. The Inv+ alpha1 box is truncated after 45 bp and is not illustrated, for details see text.
(TIF)

Table S1 ***Sclerotinia sclerotiorum*** **isolates used in this study.** Given are the isolate identifiers, the host’s scientific and common names, the location and year of collection, the source, as well as the orientation of the *MAT* inversion.
(DOC)

Table S2 ***MAT*** **gene nucleotide polymorphisms in** ***Sclerotinia sclerotiorum*** **strains 44Ba1, 44Ba12 and 44Ba18 in comparison to** ***S. sclerotiorum*** **strain 1980 (Amselem et al. 2011).**
(DOC)

Table S3 **Phylogenetic analyses of** ***Sclerotinia sclerotiorum*** **and** ***Botrytis cinerea MAT*** **genes using maximum parsimony.** For each analysis, the number of taxa, alignment length, the number of parsimony informative characters, the number and length of most parsimonious trees (MPT) and the consistency (CI) and retention indices (RI), are given. Respective alignments are available as Alignments S6, S7, S8 and S9.
(DOC)

Table S4 **Length comparisons of homologous** ***MAT*** **intergenic spacer regions in** ***Sclerotinia sclerotiorum*** **strains 44Ba1, 44Ba12, 44Ba18 and** ***S. sclerotiorum*** **strain 1980** [**17**] **.**
(DOC)

Table S5 **Top blast matches at GenBank against the 250-bp** ***Sclerotinia sclerotiorum*** **motif query using blastn with e-values equal or smaller than 0.33, only a single representative match for each gene in each species is listed.**
(DOC)

Table S6 **Orientation of** ***MAT*** **inversion region among progeny of four different** ***Sclerotinia sclerotiorum*** **parental strains, 18 to 20 progeny derived from a single apothecium were screened for each parent.** For details on parental isolates, see Table S1.
(DOCX)

Table S7 **Formation of apothecial stalks in 38** ***Sclerotinia sclerotiorum*** **isolates, boldface highlights absence of apothecial stalk formation.**
(DOC)

Table S8 **Primers used for PCR and sequencing of the** ***Sclerotinia sclerotiorum*** **mating type locus in isolates 44Ba1, 44Ba12 and 44Ba18.** The last letter in a primer name indicates the primer direction, forward and reverse, respectively.
(DOC)

Table S9 **Sequencing primers targeting the** ***MAT*** **inversion in** ***Sclerotinia sclerotiorum*** **strains 44Ba12 and 44Ba18.** The last letter in a primer name indicates the primer direction, forward and reverse, respectively.
(DOC)

Table S10 **Sequencing primers used in** ***Sclerotinia sclerotiorum*** **strain 44Ba1 targeting the** ***MAT*** **inversion region in** ***S. sclerotiorum*** **strains 44Ba12 and 44Ba18.** The last letter in a primer name indicates the primer direction, forward and reverse, respectively.
(DOC)

Table S11 **Primer pairs used for** ***Sclerotinia sclerotiorum*** **Inv+ PCR screening, and PCR amplification and DNA sequencing of inversion breakpoints and** ***MAT*** **inversion region.** The ‘f’ or ‘r’ in a primer name indicates the primer direction, forward and reverse, respectively.
(DOC)

Table S12 **Primers used for** ***Sclerotinia sclerotiorum MAT*** **gene expression analysis.**
(DOC)

Alignment S1 **FASTA text file with alignment of** ***Sclerotinia sclerotiorum*** **strains 1980, 44Ba1, 44Ba12 and 44Ba18** ***MAT*** **regions, the** ***MAT1-1-1*** **and** ***MAT1-2-1*** **transcript variants, and the** ***MAT*** **inversion region sequences of the tetrad progeny** ***S. sclerotiorum*** **strains 1B331-1 (Inv+) and 1B331-3 (Inv-) obtained with primers SS1f / SS2r.** Sequence accession numbers are given as part of sequence names. Indicated are genes, the *MAT* inversion region and the 250-bp motifs. Sequence accession numbers are given as part of sequence names for sequences in public databases.
(TXT)

Alignment S2 **FASTA text file with alignment of** ***Sclerotinia sclerotiorum*** **and** ***Botrytis cinerea MAT1-1-1*** **and** ***MAT1-2-1*** **demonstrating the absence of the 250-bp motifs in** ***B. cinerea MAT1-2-1.*** Sequence accession numbers are given as part of sequence names.
(TXT)

Alignment S3 **FASTA text file with alignment of** ***Sclerotinia sclerotiorum MAT1-1*** **with** ***Botrytis cinerea MAT1-1*** **and** ***B. cinerea MAT1-2*** **.** The potential crossover site between ancestral *MAT1-1* and *MAT1-2* is marked and includes the positions where alignment between *S. sclerotiorum* and *B. cinerea MAT1-1* is no longer possible, and a short stretch of *B. cinerea MAT1-2* region aligns. Sequence accession numbers are given as part of sequence names.
(TXT)

Alignment S4 **FASTA text file with alignment of** ***Sclerotinia sclerotiorum*** **strains 44Ba1 (Inv-), 44Ba12 (Inv+), 44Ba18 (Inv+) and 1980 (Inv-) and inversion breakpoint sequences obtained with primers SS1f, SS1r2, SS2f2 and SS2r (Table S11, Figure S1) for 32** ***S. sclerotiorum*** **isolates demonstrating that the 250-bp motifs spanning the inversion breakpoints were identical in all isolates.** Positions of strain 44Ba1 *MAT1-1-1* and *MAT1-2-1* and the 250-bp motifs are indicated. Sequence accession numbers, strain identifiers used in this study (Table S1, Table S6), and primers used to generate each sequence are part of the sequence names. None of the SS1f, SS1r2, SS2f2 or SS2r sequences was submitted to GenBank.
(TXT)

Alignment S5 **FASTA text file with alignment of all 250-bp motifs from Alignment S4, used to generate** **Figure 11** **.** The *MAT1-1-5* distal 250-bp motifs were reverse complemented before alignment. Taxon labels are as in Figure 11.
(TXT)

Alignment S6 **FASTA text file with alignment of** ***Botrytis cinerea*** **and** ***Sclerotinia sclerotiorum*** **strain** ***MAT1-1-1*** **sequences used in phylogenetic analyses for** **Figure 2** **.** Accession numbers are part of taxon names.
(TXT)

Alignment S7 **FASTA text file with alignment of** ***Botrytis cinerea*** **and** ***Sclerotinia sclerotiorum*** **strain** ***MAT1-1-5*** **sequences used in phylogenetic analyses for** **Figure 2** **.** Accession numbers are part of taxon names.
(TXT)

Alignment S8 **FASTA text file with alignment of** ***Botrytis cinerea*** **and** ***Sclerotinia sclerotiorum*** **strain** ***MAT1-2-1*** **sequences used in phylogenetic analyses for** **Figure 2** **.** Accession numbers are part of taxon names.
(TXT)

Alignment S9 **FASTA text file with alignment of** ***Botrytis cinerea*** **and** ***Sclerotinia sclerotiorum*** **strain** ***MAT1-2-4*** **sequences used in phylogenetic analyses for** **Figure 2** **.** Accession numbers are part of taxon names.
(TXT)
