# Supplementary figures and images for: The Sclerotinia sclerotiorum Mating Type Locus (MAT) Contains a 3.6-kb Region That Is Inverted in Every Meiotic Generation
Source: PLoS One. 2013 Feb 15;8(2):e56895. doi: 10.1371/journal.pone.0056895 (PMC3574095; doi:10.1371/journal.pone.0056895)

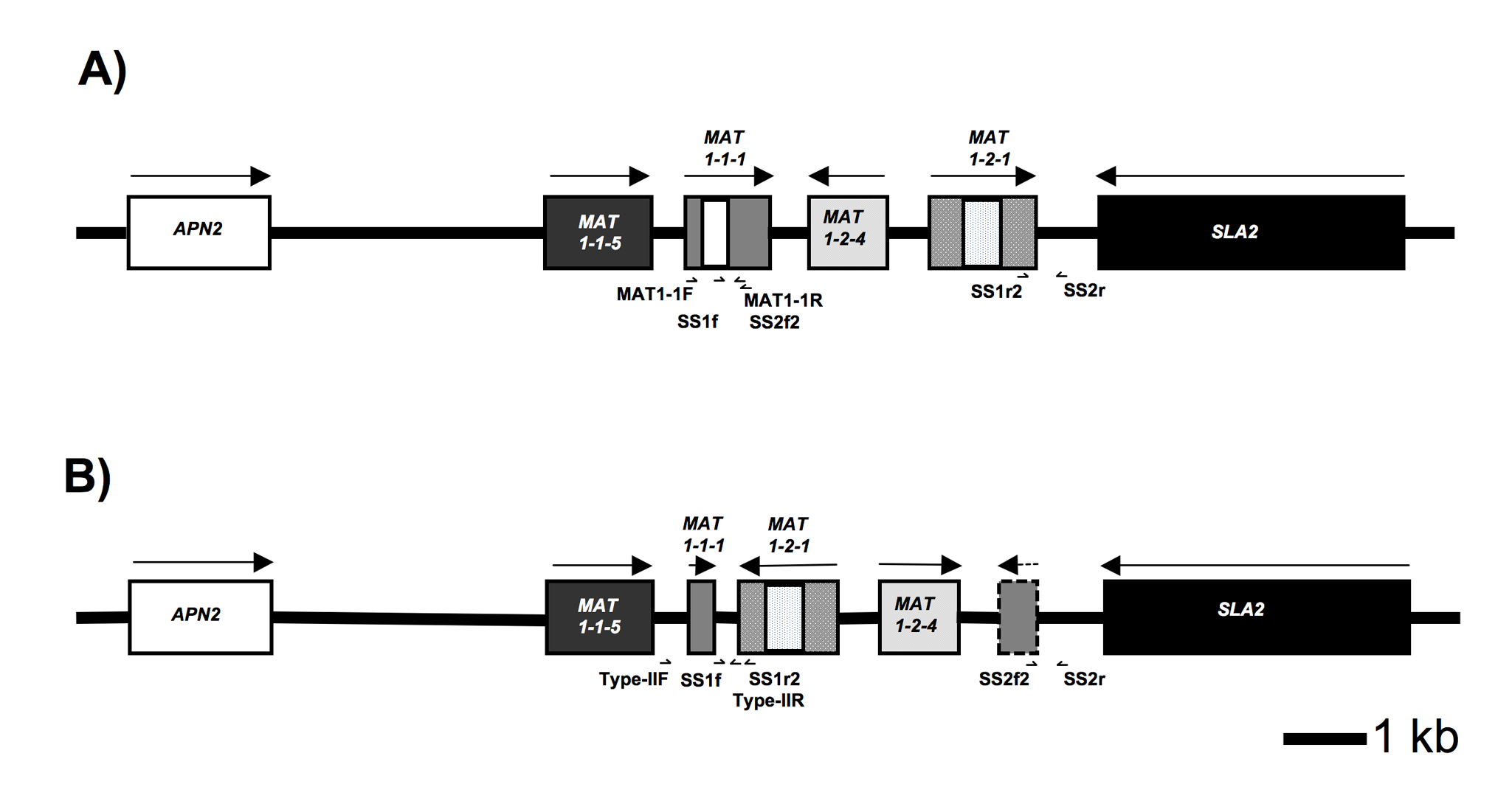

Supplement: Figure S1 — Positions of primers used for MAT inversion screening (MAT1-1F / MAT1-1R, Type-IIF / Type-IIR), and PCR amplification and sequencing of the inversion breakpoints in S. sclerotiorum isolates S1A) without (Inv-) and, S1B) with inversion (Inv+). Genes are boxes, white and dotted boxes correspond to alpha1 and HMG domains, respectively, directions of transcription are indicated by arrows, gene names are inside or by the boxes. Dashed box and arrow represent MAT1-1-1 3’-end fragment lacking an in frame start codon. Primer sites are indicated by half arrows. Diagrams are to scale. The Inv+ alpha1 box is truncated after 45 bp and is not illustrated, for details see text. (TIF) [file pone.0056895.s001.tif]
