# Supplementary material for: The Sclerotinia sclerotiorum Mating Type Locus (MAT) Contains a 3.6-kb Region That Is Inverted in Every Meiotic Generation
Source: PLoS One. 2013 Feb 15;8(2):e56895. doi: 10.1371/journal.pone.0056895 (PMC3574095; doi:10.1371/journal.pone.0056895)
Supplement: Table S1 — Sclerotinia sclerotiorum isolates used in this study. Given are the isolate identifiers, the host’s scientific and common names, the location and year of collection, the source, as well as the orientation of the MAT inversion. (DOC) [file pone.0056895.s002.doc]

Table S1. *Sclerotinia sclerotiorum* isolates used in this study. Given are the isolate identifiers, the host’s scientific and common names, the location and year of collection, the source, as well as the orientation of the *MAT* inversion region.

| **Isolate identifier** | **Host scientific name** | **Host common name** | **Location** | **Collection year** | **Source** | ***MAT* inversion region** |
| --- | --- | --- | --- | --- | --- | --- |
| BS001^A^ | *Lactuca sativa* L. | Lettuce | Salinas, CA | 1999 | Subbarao Lab Collection | Inv- |
| BS002 | *Lactuca sativa* L. | Lettuce | Salinas, CA | 1999 | Subbarao Lab Collection | Inv- |
| BS003 | *Lactuca sativa* L. | Lettuce | Salinas, CA | 1999 | Subbarao Lab Collection | Inv- |
| BS009 | *Phaseolus vulgaris* L. | Dry bean | Salinas, CA | 1999 | Subbarao Lab Collection | Inv+ |
| BS010 | *Capsicum annuum* L. | Pepper | King City, CA | 1999 | Subbarao Lab Collection | Inv- |
| BS011 | *Lactuca sativa* L. | Lettuce | Salinas, CA | 2000 | Subbarao Lab Collection | Inv- |
| BS012 | *Capsicum annuum* L. | Pepper | King City, CA | 1999 | Subbarao Lab Collection | Inv- |
| BS013 | *Lactuca sativa* L. | Lettuce | Santa Maria, CA | 1994 | Subbarao Lab Collection | Inv+ |
| BS014^A^ | *Brassica oleracea* var*. botrytis* | Cauliflower | Santa Maria, CA | 1994 | Subbarao Lab Collection | Inv+ |
| BS015 | *Lactuca sativa* L. | Lettuce | Santa Maria, CA | 1995 | Subbarao Lab Collection | Inv+ |
| BS016 | *Lactuca sativa* L. | Lettuce | Salinas, CA | 1999 | Subbarao Lab Collection | Inv+ |
| BS017 | *Lactuca sativa* L. | Lettuce | Salinas, CA | 1999 | Subbarao Lab Collection | Inv- |
| BS018 | *Lactuca sativa* L. | Lettuce | Salinas, CA | 1999 | Subbarao Lab Collection | Inv+ |
| BS019 | *Lactuca sativa* L. | Lettuce | Salinas, CA | 1999 | Subbarao Lab Collection | Inv+ |
| BS020 | *Lactuca sativa* L. | Lettuce | Huron, CA | 1999 | Subbarao Lab Collection | Inv+ |
| BS021 | *Lactuca sativa* L. | Lettuce | Huron, CA | 1999 | Subbarao Lab Collection | Inv+ |
| BS022 | *Lactuca sativa* L. | Lettuce | Huron, CA | 1999 | Subbarao Lab Collection | Inv+ |
| BS023 | *Brassica oleracea* var*. botrytis* | Cauliflower | Santa Maria, CA | 1996 | Subbarao Lab Collection | Inv+ |
| BS024 | *Lactuca sativa* L. | Lettuce | Santa Maria, CA | 1998 | Subbarao Lab Collection | Inv+ |
| BS025 | *Brassica oleracea* var*. botrytis* | Cauliflower | Santa Maria, CA | 1994 | Subbarao Lab Collection | Inv+ |
| BS026 | *Lactuca sativa* L. | Lettuce | Huron, CA | 2000 | Subbarao Lab Collection | Inv+ |
| BS027 | *Lactuca sativa* L. | Lettuce | Huron, CA | 2000 | Subbarao Lab Collection | Inv+ |
| BS028 | *Lactuca sativa* L. | Lettuce | Huron, CA | 2000 | Subbarao Lab Collection | Inv+ |
| BS030 | *Lactuca sativa* L. | Lettuce | Huron, CA | 2000 | Subbarao Lab Collection | Inv+ |
| BS031 | *Lactuca sativa* L. | Lettuce | Santa Maria, CA | 2000 | Subbarao Lab Collection | Inv+ |
| BS032 | *Lactuca sativa* L. | Lettuce | King City, CA | 2000 | Subbarao Lab Collection | Inv- |
| BS033 | *Lactuca sativa* L. | Lettuce | King City, CA | 2000 | Subbarao Lab Collection | Inv- |
| BS034 | *Lactuca sativa* L. | Lettuce | King City, CA | 2000 | Subbarao Lab Collection | Inv- |
| BS035 | *Lactuca sativa* L. | Lettuce | King City, CA | 2000 | Subbarao Lab Collection | Inv- |
| BS040 | *Lactuca sativa* L. | Lettuce | King City, CA | 2000 | Subbarao Lab Collection | Inv+ |
| BS041 | *Lactuca sativa* L. | Lettuce | King City, CA | 2000 | Subbarao Lab Collection | Inv+ |
| BS042 | *Lactuca sativa* L. | Lettuce | King City, CA | 2000 | Subbarao Lab Collection | Inv+ |
| BS047 | *Lactuca sativa* L. | Lettuce | King City, CA | 2000 | Subbarao Lab Collection | Inv+ |
| BS050 | *Lactuca sativa* L. | Lettuce | Huron, CA | 2000 | Subbarao Lab Collection | Inv+ |
| BS051 | *Lactuca sativa* L. | Lettuce | Huron, CA | 2000 | Subbarao Lab Collection | Inv+ |
| BS055 | *Lactuca sativa* L. | Lettuce | Soledad, CA | 2000 | Subbarao Lab Collection | Inv+ |
| BS057 | *Lactuca sativa* L. | Lettuce | Green Field, CA | 2000 | Subbarao Lab Collection | Inv+ |
| BS058 | *Lactuca sativa* L. | Lettuce | Green Field, CA | 2000 | Subbarao Lab Collection | Inv+ |
| BS061 | *Lactuca sativa* L. | Lettuce | Green Field, CA | 2000 | Subbarao Lab Collection | Inv- |
| BS062 | *Lactuca sativa* L. | Lettuce | Green Field, CA | 2000 | Subbarao Lab Collection | Inv+ |
| BS063 | *Lactuca sativa* L. | Lettuce | Salinas, CA | 2000 | Subbarao Lab Collection | Inv+ |
| BS064 | *Lactuca sativa* L. | Lettuce | Salinas, CA | 2000 | Subbarao Lab Collection | Inv+ |
| BS065 | *Lactuca sativa* L. | Lettuce | Gonzales, CA | 2000 | Subbarao Lab Collection | Inv- |
| BS066 | *Lactuca sativa* L. | Lettuce | Gonzales, CA | 2000 | Subbarao Lab Collection | Inv- |
| BS067 | *Lactuca sativa* L. | Lettuce | Gonzales, CA | 2000 | Subbarao Lab Collection | Inv+ |
| BS068 | *Lactuca sativa* L. | Lettuce | Gonzales, CA | 2000 | Subbarao Lab Collection | Inv+ |
| BS070 | *Lactuca sativa* L. | Lettuce | Gonzales, CA | 2000 | Subbarao Lab Collection | Inv+ |
| BS071 | *Lactuca sativa* L. | Lettuce | Chular, CA | 2000 | Subbarao Lab Collection | Inv+ |
| BS076 | *Lactuca sativa* L. | Lettuce | Soledad, CA | 2000 | Subbarao Lab Collection | Inv- |
| BS079 | *Lactuca sativa* L. | Lettuce | Chular, CA | 2000 | Subbarao Lab Collection | Inv- |
| BS081 | *Lactuca sativa* L. | Lettuce | Gonzales, CA | 2000 | Subbarao Lab Collection | Inv- |
| BS082 | *Lactuca sativa* L. | Lettuce | Huron, CA | 2001 | Subbarao Lab Collection | Inv+ |
| BS083 | *Lactuca sativa* L. | Lettuce | Huron, CA | 2001 | Subbarao Lab Collection | Inv+ |
| BS084 | *Lactuca sativa* L. | Lettuce | Huron, CA | 2001 | Subbarao Lab Collection | Inv- |
| BS088 | *Lactuca sativa* L. | Lettuce | Huron, CA | 2001 | Subbarao Lab Collection | Inv- |
| BS089 | *Lactuca sativa* L. | Lettuce | Salinas, CA | 2001 | Subbarao Lab Collection | Inv- |
| BS090 | *Lactuca sativa* L. | Lettuce | Huron, CA | 2001 | Subbarao Lab Collection | Inv+ |
| BS095 | *Lactuca sativa* L. | Lettuce | Huron, CA | 2001 | Subbarao Lab Collection | Inv+ |
| BS096 | *Lactuca sativa* L. | Lettuce | Huron, CA | 2001 | Subbarao Lab Collection | Inv- |
| 321A1 | *Lactuca sativa* L. | Lettuce | San Joaquin, CA | 2008 | Subbarao Lab Collection | Inv+ |
| 321A2 | *Lactuca sativa* L. | Lettuce | San Joaquin, CA | 2008 | Subbarao Lab Collection | Inv- |
| 321A3 | *Lactuca sativa* L. | Lettuce | San Joaquin, CA | 2008 | Subbarao Lab Collection | Inv- |
| 321A4 | *Lactuca sativa* L. | Lettuce | San Joaquin, CA | 2008 | Subbarao Lab Collection | Inv+ |
| 321A5 | *Lactuca sativa* L. | Lettuce | San Joaquin, CA | 2008 | Subbarao Lab Collection | Inv- |
| 321A6 | *Lactuca sativa* L. | Lettuce | San Joaquin, CA | 2008 | Subbarao Lab Collection | Inv- |
| 321A7 | *Lactuca sativa* L. | Lettuce | San Joaquin, CA | 2008 | Subbarao Lab Collection | Inv+ |
| 321A8 | *Lactuca sativa* L. | Lettuce | San Joaquin, CA | 2008 | Subbarao Lab Collection | Inv- |
| 321A9 | *Lactuca sativa* L. | Lettuce | San Joaquin, CA | 2008 | Subbarao Lab Collection | Inv+ |
| 321A10 | *Lactuca sativa* L. | Lettuce | San Joaquin, CA | 2008 | Subbarao Lab Collection | Inv- |
| 321A11 | *Lactuca sativa* L. | Lettuce | San Joaquin, CA | 2008 | Subbarao Lab Collection | Inv+ |
| 321B1 | *Lactuca sativa* L. | Lettuce | San Joaquin, CA | 2008 | Subbarao Lab Collection | Inv- |
| 321B2 | *Lactuca sativa* L. | Lettuce | San Joaquin, CA | 2008 | Subbarao Lab Collection | Inv+ |
| 321B3 | *Lactuca sativa* L. | Lettuce | San Joaquin, CA | 2008 | Subbarao Lab Collection | Inv+ |
| 321B4 | *Lactuca sativa* L. | Lettuce | San Joaquin, CA | 2008 | Subbarao Lab Collection | Inv+ |
| 321B5 | *Lactuca sativa* L. | Lettuce | San Joaquin, CA | 2008 | Subbarao Lab Collection | Inv+ |
| 321B6 | *Lactuca sativa* L. | Lettuce | San Joaquin, CA | 2008 | Subbarao Lab Collection | Inv- |
| 321B7 | *Lactuca sativa* L. | Lettuce | San Joaquin, CA | 2008 | Subbarao Lab Collection | Inv- |
| 321B8 | *Lactuca sativa* L. | Lettuce | San Joaquin, CA | 2008 | Subbarao Lab Collection | Inv- |
| 321B9 | *Lactuca sativa* L. | Lettuce | San Joaquin, CA | 2008 | Subbarao Lab Collection | Inv- |
| 321B10 | *Lactuca sativa* L. | Lettuce | San Joaquin, CA | 2008 | Subbarao Lab Collection | Inv+ |
| 321B11 | *Lactuca sativa* L. | Lettuce | San Joaquin, CA | 2008 | Subbarao Lab Collection | Inv- |
| 321B12 | *Lactuca sativa* L. | Lettuce | San Joaquin, CA | 2008 | Subbarao Lab Collection | Inv- |
| 321B13 | *Lactuca sativa* L. | Lettuce | San Joaquin, CA | 2008 | Subbarao Lab Collection | Inv+ |
| 321B14 | *Lactuca sativa* L. | Lettuce | San Joaquin, CA | 2008 | Subbarao Lab Collection | Inv- |
| 321B15 | *Lactuca sativa* L. | Lettuce | San Joaquin, CA | 2008 | Subbarao Lab Collection | Inv- |
| 321B16 | *Lactuca sativa* L. | Lettuce | San Joaquin, CA | 2008 | Subbarao Lab Collection | Inv- |
| 321B17 | *Lactuca sativa* L. | Lettuce | San Joaquin, CA | 2008 | Subbarao Lab Collection | Inv+ |
| 321B18 | *Lactuca sativa* L. | Lettuce | San Joaquin, CA | 2008 | Subbarao Lab Collection | Inv- |
| 321B19 | *Lactuca sativa* L. | Lettuce | San Joaquin, CA | 2008 | Subbarao Lab Collection | Inv- |
| 321B20 | *Lactuca sativa* L. | Lettuce | San Joaquin, CA | 2008 | Subbarao Lab Collection | Inv+ |
| 321B21 | *Lactuca sativa* L. | Lettuce | San Joaquin, CA | 2008 | Subbarao Lab Collection | Inv+ |
| 321B22 | *Lactuca sativa* L. | Lettuce | San Joaquin, CA | 2008 | Subbarao Lab Collection | Inv- |
| 321C1 | *Lactuca sativa* L. | Lettuce | San Joaquin, CA | 2008 | Subbarao Lab Collection | Inv- |
| 321C2 | *Lactuca sativa* L. | Lettuce | San Joaquin, CA | 2008 | Subbarao Lab Collection | Inv- |
| 321C3 | *Lactuca sativa* L. | Lettuce | San Joaquin, CA | 2008 | Subbarao Lab Collection | Inv- |
| 321C4 | *Lactuca sativa* L. | Lettuce | San Joaquin, CA | 2008 | Subbarao Lab Collection | Inv- |
| 321Da1 | *Lactuca sativa* L. | Lettuce | San Joaquin, CA | 2008 | Subbarao Lab Collection | Inv+ |
| 321Da2 | *Lactuca sativa* L. | Lettuce | San Joaquin, CA | 2008 | Subbarao Lab Collection | Inv+ |
| 321Da3 | *Lactuca sativa* L. | Lettuce | San Joaquin, CA | 2008 | Subbarao Lab Collection | Inv- |
| 321Da4 | *Lactuca sativa* L. | Lettuce | San Joaquin, CA | 2008 | Subbarao Lab Collection | Inv- |
| 321Da5 | *Lactuca sativa* L. | Lettuce | San Joaquin, CA | 2008 | Subbarao Lab Collection | Inv- |
| 321Da6 | *Lactuca sativa* L. | Lettuce | San Joaquin, CA | 2008 | Subbarao Lab Collection | Inv- |
| 321Da7 | *Lactuca sativa* L. | Lettuce | San Joaquin, CA | 2008 | Subbarao Lab Collection | Inv- |
| 321Da8 | *Lactuca sativa* L. | Lettuce | San Joaquin, CA | 2008 | Subbarao Lab Collection | Inv- |
| 321Da9 | *Lactuca sativa* L. | Lettuce | San Joaquin, CA | 2008 | Subbarao Lab Collection | Inv- |
| 321Da10 | *Lactuca sativa* L. | Lettuce | San Joaquin, CA | 2008 | Subbarao Lab Collection | Inv- |
| 321Da11 | *Lactuca sativa* L. | Lettuce | San Joaquin, CA | 2008 | Subbarao Lab Collection | Inv+ |
| 321Da12 | *Lactuca sativa* L. | Lettuce | San Joaquin, CA | 2008 | Subbarao Lab Collection | Inv- |
| 321Da13 | *Lactuca sativa* L. | Lettuce | San Joaquin, CA | 2008 | Subbarao Lab Collection | Inv+ |
| 321Da14 | *Lactuca sativa* L. | Lettuce | San Joaquin, CA | 2008 | Subbarao Lab Collection | Inv+ |
| 321Da15 | *Lactuca sativa* L. | Lettuce | San Joaquin, CA | 2008 | Subbarao Lab Collection | Inv+ |
| 321Da16 | *Lactuca sativa* L. | Lettuce | San Joaquin, CA | 2008 | Subbarao Lab Collection | Inv+ |
| 321Da17 | *Lactuca sativa* L. | Lettuce | San Joaquin, CA | 2008 | Subbarao Lab Collection | Inv- |
| 321Da18 | *Lactuca sativa* L. | Lettuce | San Joaquin, CA | 2008 | Subbarao Lab Collection | Inv+ |
| 321Da19 | *Lactuca sativa* L. | Lettuce | San Joaquin, CA | 2008 | Subbarao Lab Collection | Inv+ |
| 321Da20 | *Lactuca sativa* L. | Lettuce | San Joaquin, CA | 2008 | Subbarao Lab Collection | Inv+ |
| 321Da21 | *Lactuca sativa* L. | Lettuce | San Joaquin, CA | 2008 | Subbarao Lab Collection | Inv+ |
| 321Db1 | *Lactuca sativa* L. | Lettuce | San Joaquin, CA | 2008 | Subbarao Lab Collection | Inv- |
| 321Db2 | *Lactuca sativa* L. | Lettuce | San Joaquin, CA | 2008 | Subbarao Lab Collection | Inv- |
| 321Db3 | *Lactuca sativa* L. | Lettuce | San Joaquin, CA | 2008 | Subbarao Lab Collection | Inv+ |
| 321Db4 | *Lactuca sativa* L. | Lettuce | San Joaquin, CA | 2008 | Subbarao Lab Collection | Inv+ |
| 321Db5 | *Lactuca sativa* L. | Lettuce | San Joaquin, CA | 2008 | Subbarao Lab Collection | Inv- |
| 321Db6 | *Lactuca sativa* L. | Lettuce | San Joaquin, CA | 2008 | Subbarao Lab Collection | Inv- |
| 321Db7 | *Lactuca sativa* L. | Lettuce | San Joaquin, CA | 2008 | Subbarao Lab Collection | Inv- |
| 321Db8 | *Lactuca sativa* L. | Lettuce | San Joaquin, CA | 2008 | Subbarao Lab Collection | Inv+ |
| 321Db9 | *Lactuca sativa* L. | Lettuce | San Joaquin, CA | 2008 | Subbarao Lab Collection | Inv- |
| 321Db10 | *Lactuca sativa* L. | Lettuce | San Joaquin, CA | 2008 | Subbarao Lab Collection | Inv- |
| 321Db11 | *Lactuca sativa* L. | Lettuce | San Joaquin, CA | 2008 | Subbarao Lab Collection | Inv- |
| 321Db12 | *Lactuca sativa* L. | Lettuce | San Joaquin, CA | 2008 | Subbarao Lab Collection | Inv- |
| 321Db13 | *Lactuca sativa* L. | Lettuce | San Joaquin, CA | 2008 | Subbarao Lab Collection | Inv- |
| 321Db14 | *Lactuca sativa* L. | Lettuce | San Joaquin, CA | 2008 | Subbarao Lab Collection | Inv- |
| 321Db15 | *Lactuca sativa* L. | Lettuce | San Joaquin, CA | 2008 | Subbarao Lab Collection | Inv+ |
| 321Db16 | *Lactuca sativa* L. | Lettuce | San Joaquin, CA | 2008 | Subbarao Lab Collection | Inv- |
| 321Db17 | *Lactuca sativa* L. | Lettuce | San Joaquin, CA | 2008 | Subbarao Lab Collection | Inv- |
| 321Db18 | *Lactuca sativa* L. | Lettuce | San Joaquin, CA | 2008 | Subbarao Lab Collection | Inv- |
| 321Db19 | *Lactuca sativa* L. | Lettuce | San Joaquin, CA | 2008 | Subbarao Lab Collection | Inv+ |
| 44Ba1 | *Lactuca sativa* L. | Lettuce | San Joaquin, CA | 2008 | Subbarao Lab Collection | Inv- |
| 44Ba2 | *Lactuca sativa* L. | Lettuce | San Joaquin, CA | 2008 | Subbarao Lab Collection | Inv- |
| 44Ba3 | *Lactuca sativa* L. | Lettuce | San Joaquin, CA | 2008 | Subbarao Lab Collection | Inv- |
| 44Ba4 | *Lactuca sativa* L. | Lettuce | San Joaquin, CA | 2008 | Subbarao Lab Collection | Inv+ |
| 44Ba5 | *Lactuca sativa* L. | Lettuce | San Joaquin, CA | 2008 | Subbarao Lab Collection | Inv- |
| 44Ba6 | *Lactuca sativa* L. | Lettuce | San Joaquin, CA | 2008 | Subbarao Lab Collection | Inv- |
| 44Ba7 | *Lactuca sativa* L. | Lettuce | San Joaquin, CA | 2008 | Subbarao Lab Collection | Inv+ |
| 44Ba8 | *Lactuca sativa* L. | Lettuce | San Joaquin, CA | 2008 | Subbarao Lab Collection | Inv+ |
| 44Ba9 | *Lactuca sativa* L. | Lettuce | San Joaquin, CA | 2008 | Subbarao Lab Collection | Inv+ |
| 44Ba10 | *Lactuca sativa* L. | Lettuce | San Joaquin, CA | 2008 | Subbarao Lab Collection | Inv- |
| 44Ba11 | *Lactuca sativa* L. | Lettuce | San Joaquin, CA | 2008 | Subbarao Lab Collection | Inv- |
| 44Ba12 | *Lactuca sativa* L. | Lettuce | San Joaquin, CA | 2008 | Subbarao Lab Collection | Inv+ |
| 44Ba13 | *Lactuca sativa* L. | Lettuce | San Joaquin, CA | 2008 | Subbarao Lab Collection | Inv- |
| 44Ba14 | *Lactuca sativa* L. | Lettuce | San Joaquin, CA | 2008 | Subbarao Lab Collection | Inv+ |
| 44Ba15 | *Lactuca sativa* L. | Lettuce | San Joaquin, CA | 2008 | Subbarao Lab Collection | Inv+ |
| 44Ba16 | *Lactuca sativa* L. | Lettuce | San Joaquin, CA | 2008 | Subbarao Lab Collection | Inv- |
| 44Ba17 | *Lactuca sativa* L. | Lettuce | San Joaquin, CA | 2008 | Subbarao Lab Collection | Inv- |
| 44Ba18 | *Lactuca sativa* L. | Lettuce | San Joaquin, CA | 2008 | Subbarao Lab Collection | Inv+ |
| 44Bb1 | *Lactuca sativa* L. | Lettuce | San Joaquin, CA | 2008 | Subbarao Lab Collection | Inv+ |
| 44Bb2 | *Lactuca sativa* L. | Lettuce | San Joaquin, CA | 2008 | Subbarao Lab Collection | Inv- |
| 44Bb3 | *Lactuca sativa* L. | Lettuce | San Joaquin, CA | 2008 | Subbarao Lab Collection | Inv- |
| 44Bb4 | *Lactuca sativa* L. | Lettuce | San Joaquin, CA | 2008 | Subbarao Lab Collection | Inv- |
| 44Bb5 | *Lactuca sativa* L. | Lettuce | San Joaquin, CA | 2008 | Subbarao Lab Collection | Inv+ |
| 44Bb6 | *Lactuca sativa* L. | Lettuce | San Joaquin, CA | 2008 | Subbarao Lab Collection | Inv- |
| 44Bb7 | *Lactuca sativa* L. | Lettuce | San Joaquin, CA | 2008 | Subbarao Lab Collection | Inv- |
| 44Bb8 | *Lactuca sativa* L. | Lettuce | San Joaquin, CA | 2008 | Subbarao Lab Collection | Inv- |
| 44Bb9 | *Lactuca sativa* L. | Lettuce | San Joaquin, CA | 2008 | Subbarao Lab Collection | Inv- |
| 44Bb10 | *Lactuca sativa* L. | Lettuce | San Joaquin, CA | 2008 | Subbarao Lab Collection | Inv- |
| 44Bb11 | *Lactuca sativa* L. | Lettuce | San Joaquin, CA | 2008 | Subbarao Lab Collection | Inv+ |
| 44Bb12 | *Lactuca sativa* L. | Lettuce | San Joaquin, CA | 2008 | Subbarao Lab Collection | Inv+ |
| 44Bb13 | *Lactuca sativa* L. | Lettuce | San Joaquin, CA | 2008 | Subbarao Lab Collection | Inv+ |
| 44Bb14 | *Lactuca sativa* L. | Lettuce | San Joaquin, CA | 2008 | Subbarao Lab Collection | Inv+ |
| 44Bb15 | *Lactuca sativa* L. | Lettuce | San Joaquin, CA | 2008 | Subbarao Lab Collection | Inv+ |
| 44Bb16 | *Lactuca sativa* L. | Lettuce | San Joaquin, CA | 2008 | Subbarao Lab Collection | Inv- |
| 44Bb17 | *Lactuca sativa* L. | Lettuce | San Joaquin, CA | 2008 | Subbarao Lab Collection | Inv+ |
| 44Bb18 | *Lactuca sativa* L. | Lettuce | San Joaquin, CA | 2008 | Subbarao Lab Collection | Inv- |
| 44Bb19 | *Lactuca sativa* L. | Lettuce | San Joaquin, CA | 2008 | Subbarao Lab Collection | Inv- |
| 44Bb20 | *Lactuca sativa* L. | Lettuce | San Joaquin, CA | 2008 | Subbarao Lab Collection | Inv+ |
| 44Bb21 | *Lactuca sativa* L. | Lettuce | San Joaquin, CA | 2008 | Subbarao Lab Collection | Inv+ |
| 44Bb22 | *Lactuca sativa* L. | Lettuce | San Joaquin, CA | 2008 | Subbarao Lab Collection | Inv- |
| 44Ea1 | *Lactuca sativa* L. | Lettuce | San Joaquin, CA | 2008 | Subbarao Lab Collection | Inv- |
| 44Ea2 | *Lactuca sativa* L. | Lettuce | San Joaquin, CA | 2008 | Subbarao Lab Collection | Inv+ |
| 44Ea3 | *Lactuca sativa* L. | Lettuce | San Joaquin, CA | 2008 | Subbarao Lab Collection | Inv+ |
| 44Ea4 | *Lactuca sativa* L. | Lettuce | San Joaquin, CA | 2008 | Subbarao Lab Collection | Inv- |
| 44Ea5 | *Lactuca sativa* L. | Lettuce | San Joaquin, CA | 2008 | Subbarao Lab Collection | Inv+ |
| 44Ea6 | *Lactuca sativa* L. | Lettuce | San Joaquin, CA | 2008 | Subbarao Lab Collection | Inv- |
| 44Ea7 | *Lactuca sativa* L. | Lettuce | San Joaquin, CA | 2008 | Subbarao Lab Collection | Inv- |
| 44Ea8 | *Lactuca sativa* L. | Lettuce | San Joaquin, CA | 2008 | Subbarao Lab Collection | Inv+ |
| 44Ea9 | *Lactuca sativa* L. | Lettuce | San Joaquin, CA | 2008 | Subbarao Lab Collection | Inv+ |
| 44Ea10 | *Lactuca sativa* L. | Lettuce | San Joaquin, CA | 2008 | Subbarao Lab Collection | Inv- |
| 44Ea11 | *Lactuca sativa* L. | Lettuce | San Joaquin, CA | 2008 | Subbarao Lab Collection | Inv- |
| 44Ea12 | *Lactuca sativa* L. | Lettuce | San Joaquin, CA | 2008 | Subbarao Lab Collection | Inv+ |
| 44Ea13 | *Lactuca sativa* L. | Lettuce | San Joaquin, CA | 2008 | Subbarao Lab Collection | Inv- |
| 44Ea14 | *Lactuca sativa* L. | Lettuce | San Joaquin, CA | 2008 | Subbarao Lab Collection | Inv- |
| 44Ea15 | *Lactuca sativa* L. | Lettuce | San Joaquin, CA | 2008 | Subbarao Lab Collection | Inv- |
| 44Ea16 | *Lactuca sativa* L. | Lettuce | San Joaquin, CA | 2008 | Subbarao Lab Collection | Inv- |
| 44Ea17 | *Lactuca sativa* L. | Lettuce | San Joaquin, CA | 2008 | Subbarao Lab Collection | Inv- |
| 44Ea18 | *Lactuca sativa* L. | Lettuce | San Joaquin, CA | 2008 | Subbarao Lab Collection | Inv- |
| 44Ea19 | *Lactuca sativa* L. | Lettuce | San Joaquin, CA | 2008 | Subbarao Lab Collection | Inv- |
| 44Ea20 | *Lactuca sativa* L. | Lettuce | San Joaquin, CA | 2008 | Subbarao Lab Collection | Inv- |
| 44Ea21 | *Lactuca sativa* L. | Lettuce | San Joaquin, CA | 2008 | Subbarao Lab Collection | Inv- |
| 44Ea22 | *Lactuca sativa* L. | Lettuce | San Joaquin, CA | 2008 | Subbarao Lab Collection | Inv- |
| 44Ea23 | *Lactuca sativa* L. | Lettuce | San Joaquin, CA | 2008 | Subbarao Lab Collection | Inv- |
| 44Ea24 | *Lactuca sativa* L. | Lettuce | San Joaquin, CA | 2008 | Subbarao Lab Collection | Inv- |
| 44Ea25 | *Lactuca sativa* L. | Lettuce | San Joaquin, CA | 2008 | Subbarao Lab Collection | Inv+ |
| 44Ea26 | *Lactuca sativa* L. | Lettuce | San Joaquin, CA | 2008 | Subbarao Lab Collection | Inv- |
| 44Eb1 | *Lactuca sativa* L. | Lettuce | San Joaquin, CA | 2008 | Subbarao Lab Collection | Inv- |
| 44Eb2 | *Lactuca sativa* L. | Lettuce | San Joaquin, CA | 2008 | Subbarao Lab Collection | Inv- |
| 44Eb3 | *Lactuca sativa* L. | Lettuce | San Joaquin, CA | 2008 | Subbarao Lab Collection | Inv- |
| 44Eb4 | *Lactuca sativa* L. | Lettuce | San Joaquin, CA | 2008 | Subbarao Lab Collection | Inv- |
| 44Eb5 | *Lactuca sativa* L. | Lettuce | San Joaquin, CA | 2008 | Subbarao Lab Collection | Inv- |
| 44Eb6 | *Lactuca sativa* L. | Lettuce | San Joaquin, CA | 2008 | Subbarao Lab Collection | Inv- |
| 44Eb7 | *Lactuca sativa* L. | Lettuce | San Joaquin, CA | 2008 | Subbarao Lab Collection | Inv- |
| 44Eb8 | *Lactuca sativa* L. | Lettuce | San Joaquin, CA | 2008 | Subbarao Lab Collection | Inv+ |
| 44Eb9 | *Lactuca sativa* L. | Lettuce | San Joaquin, CA | 2008 | Subbarao Lab Collection | Inv- |
| 44Eb10 | *Lactuca sativa* L. | Lettuce | San Joaquin, CA | 2008 | Subbarao Lab Collection | Inv- |
| 44Eb11 | *Lactuca sativa* L. | Lettuce | San Joaquin, CA | 2008 | Subbarao Lab Collection | Inv- |
| 44Eb12 | *Lactuca sativa* L. | Lettuce | San Joaquin, CA | 2008 | Subbarao Lab Collection | Inv- |
| 44Eb13 | *Lactuca sativa* L. | Lettuce | San Joaquin, CA | 2008 | Subbarao Lab Collection | Inv+ |
| 44Eb14 | *Lactuca sativa* L. | Lettuce | San Joaquin, CA | 2008 | Subbarao Lab Collection | Inv+ |
| 44Eb15 | *Lactuca sativa* L. | Lettuce | San Joaquin, CA | 2008 | Subbarao Lab Collection | Inv+ |
| 44Eb16 | *Lactuca sativa* L. | Lettuce | San Joaquin, CA | 2008 | Subbarao Lab Collection | Inv+ |
| 44Eb17 | *Lactuca sativa* L. | Lettuce | San Joaquin, CA | 2008 | Subbarao Lab Collection | Inv- |
| 44Eb18 | *Lactuca sativa* L. | Lettuce | San Joaquin, CA | 2008 | Subbarao Lab Collection | Inv+ |
| 1B321-2^B^ | NA | NA | NA | 2002 | Subbarao Lab Collection | Inv+ |
| 1B321-4^B^ | NA | NA | NA | 2002 | Subbarao Lab Collection | Inv+ |
| 1B321-6^B^ | NA | NA | NA | 2002 | Subbarao Lab Collection | Inv- |
| 1B321-8^B^ | NA | NA | NA | 2002 | Subbarao Lab Collection | Inv- |
| 1B331-1^C^ | NA | NA | NA | 2002 | Subbarao Lab Collection | Inv+ |
| 1B331-2^C^ | NA | NA | NA | 2002 | Subbarao Lab Collection | Inv+ |
| 1B331-3^C^ | NA | NA | NA | 2002 | Subbarao Lab Collection | Inv- |
| 1B331-4^C^ | NA | NA | NA | 2002 | Subbarao Lab Collection | Inv- |
| 1B331-5^C^ | NA | NA | NA | 2002 | Subbarao Lab Collection | Inv+ |
| 1B331-6^C^ | NA | NA | NA | 2002 | Subbarao Lab Collection | Inv+ |
| 1B331-7^C^ | NA | NA | NA | 2002 | Subbarao Lab Collection | Inv- |
| 1B331-8^C^ | NA | NA | NA | 2002 | Subbarao Lab Collection | Inv- |
| 570 | *Phaseolus vulgaris* L. | Dry bean | Scottsbluff, NE | 2004 | Steadman Lab Collection | Inv+ |
| 572 | *Phaseolus vulgaris* L. | Dry bean | Scottsbluff, NE | 2004 | Steadman Lab Collection | Inv+ |
| 574 | *Phaseolus vulgaris* L. | Dry bean | Scottsbluff, NE | 2004 | Steadman Lab Collection | Inv+ |
| 576 | *Phaseolus vulgaris* L. | Dry bean | Scottsbluff, NE | 2004 | Steadman Lab Collection | Inv+ |
| 578 | *Phaseolus vulgaris* L. | Dry bean | Scottsbluff, NE | 2004 | Steadman Lab Collection | Inv- |
| 636 | *Phaseolus vulgaris* L. | Dry bean | Mitchell, NE | 2005 | Steadman Lab Collection | Inv- |
| 638 | *Phaseolus vulgaris* L. | Dry bean | Mitchell, NE | 2005 | Steadman Lab Collection | Inv- |
| 640 | *Phaseolus vulgaris* L. | Dry bean | Mitchell, NE | 2005 | Steadman Lab Collection | Inv+ |
| 641 | *Phaseolus vulgaris* L. | Dry bean | Mitchell, NE | 2005 | Steadman Lab Collection | Inv+ |
| 642 | *Phaseolus vulgaris* L. | Dry bean | Mitchell, NE | 2005 | Steadman Lab Collection | Inv- |
| 644 | *Phaseolus vulgaris* L. | Dry bean | Mitchell, NE | 2005 | Steadman Lab Collection | Inv+ |
| 746 | *Phaseolus vulgaris* L. | Dry bean | Mitchell, NE | 2007 | Steadman Lab Collection | Inv+ |
| 748 | *Phaseolus vulgaris* L. | Dry bean | Mitchell, NE | 2007 | Steadman Lab Collection | Inv+ |
| 750 | *Phaseolus vulgaris* L. | Dry bean | Mitchell, NE | 2007 | Steadman Lab Collection | Inv+ |
| 752 | *Phaseolus vulgaris* L. | Dry bean | Scottsbluff, NE | 2007 | Steadman Lab Collection | Inv+ |
| 754 | *Phaseolus vulgaris* L. | Dry bean | Scottsbluff, NE | 2007 | Steadman Lab Collection | Inv+ |
| 824 | - | Dry bean field soil | Scottsbluff, NE | 2008 | Steadman Lab Collection | Inv- |
| 826 | - | Dry bean field soil | Scottsbluff, NE | 2008 | Steadman Lab Collection | Inv+ |
| 827 | - | Dry bean field soil | Scottsbluff, NE | 2008 | Steadman Lab Collection | Inv+ |
| 828 | - | Dry bean field soil | Scottsbluff, NE | 2008 | Steadman Lab Collection | Inv- |
| 830 | - | Dry bean field soil | Scottsbluff, NE | 2008 | Steadman Lab Collection | Inv+ |
| 832 | - | Dry bean field soil | Scottsbluff, NE | 2008 | Steadman Lab Collection | Inv+ |
| 858 | *Phaseolus vulgaris* L. | Dry bean | Mitchell, NE | 2010 | Steadman Lab Collection | Inv- |
| 860 | *Phaseolus vulgaris* L. | Dry bean | Mitchell, NE | 2010 | Steadman Lab Collection | Inv- |
| 862 | *Phaseolus vulgaris* L. | Dry bean | Mitchell, NE | 2010 | Steadman Lab Collection | Inv- |
| 727 | *Brassica napus* L*.* | Canola | GA | 1992 | Phillip Lab Collection | Inv- |
| 728 | *Brassica napus* L*.* | Canola | GA | 1992 | Phillip Lab Collection | Inv+ |
| 136 | *Helianthus annuus* L. | Sunflower | MN | 2008 | Nelson Lab Collection | Inv- |
| 148 | *Glycine max* (L.) Merr. | Soybean | IL | 2008 | Nelson Lab Collection | Inv- |
| 156 | *Phaseolus vulgaris* L. | Dry bean | ND | 2008 | Nelson Lab Collection | Inv+ |
| 191 | *Glycine max* (L.) Merr. | Soybean | NE | 2008 | Nelson Lab Collection | Inv- |
| 201 | *Glycine max* (L.) Merr. | Soybean | SD | 2008 | Nelson Lab Collection | Inv+ |
| 210 | *Glycine max* (L.) Merr. | Soybean | OH | 2002 | Nelson Lab Collection | Inv+ |
| 232 | *Nicotiana tabacum* L. | Tobacco | WI | 2003 | Nelson Lab Collection | Inv- |
| 234 | *Glycine max* (L.) Merr. | Soybean | MO | 2004 | Nelson Lab Collection | Inv+ |
| 246 | *Helianthus annuus* L. | Sunflower | KS | 2008 | Nelson Lab Collection | Inv- |
| 2 | *Solanum tuberosum* L. | Potato | Columbia Basin, WA | 2000-2002 | Johnson Lab Collection | Inv+ |
| 11 | *Solanum tuberosum* L. | Potato | Columbia Basin, WA | 2000-2002 | Johnson Lab Collection | Inv+ |
| 12 | *Solanum tuberosum* L. | Potato | Columbia Basin, WA | 2000-2002 | Johnson Lab Collection | Inv+ |
| 13 | *Solanum tuberosum* L. | Potato | Columbia Basin, WA | 2000-2002 | Johnson Lab Collection | Inv+ |
| 31 | *Solanum tuberosum* L. | Potato | Columbia Basin, WA | 2000-2002 | Johnson Lab Collection | Inv+ |
| 32 | *Solanum tuberosum* L. | Potato | Columbia Basin, WA | 2000-2002 | Johnson Lab Collection | Inv+ |
| 41 | *Solanum tuberosum* L. | Potato | Columbia Basin, WA | 2000-2002 | Johnson Lab Collection | Inv+ |
| 42 | *Solanum tuberosum* L. | Potato | Columbia Basin, WA | 2000-2002 | Johnson Lab Collection | Inv+ |
| 43 | *Solanum tuberosum* L. | Potato | Columbia Basin, WA | 2000-2002 | Johnson Lab Collection | Inv+ |
| 51 | *Solanum tuberosum* L. | Potato | Columbia Basin, WA | 2000-2002 | Johnson Lab Collection | Inv+ |
| 52 | *Solanum tuberosum* L. | Potato | Columbia Basin, WA | 2000-2002 | Johnson Lab Collection | Inv+ |
| 53 | *Solanum tuberosum* L. | Potato | Columbia Basin, WA | 2000-2002 | Johnson Lab Collection | Inv+ |
| 61 | *Solanum tuberosum* L. | Potato | Columbia Basin, WA | 2000-2002 | Johnson Lab Collection | Inv+ |
| 62 | *Solanum tuberosum* L. | Potato | Columbia Basin, WA | 2000-2002 | Johnson Lab Collection | Inv+ |
| 63 | *Solanum tuberosum* L. | Potato | Columbia Basin, WA | 2000-2002 | Johnson Lab Collection | Inv+ |

^A^ One of the two isolates that are in contention for tetrad parent, our notes were inconclusive which one of the two strains was used.

^B^ Isolate is part of the incomplete ordered tetrad generated from *S. sclerotiorum* strain BS001 or BS014 in the laboratory, the last digit of the strain identifier indicates the ascospore position in the ascus (from top to bottom).

^C^ Isolate is part of the complete ordered tetrad generated from *S. sclerotiorum* strain BS001 or BS014 in the laboratory, the last digit of the strain identifier indicates the ascospore position in the ascus (from top to bottom).
