# Supplementary material for: The Sclerotinia sclerotiorum Mating Type Locus (MAT) Contains a 3.6-kb Region That Is Inverted in Every Meiotic Generation
Source: PLoS One. 2013 Feb 15;8(2):e56895. doi: 10.1371/journal.pone.0056895 (PMC3574095; doi:10.1371/journal.pone.0056895)
Supplement: Table S2 — MAT gene nucleotide polymorphisms in Sclerotinia sclerotiorum strains 44Ba1, 44Ba12 and 44Ba18 in comparison to S. sclerotiorum strain 1980 (Amselem et al. 2011). (DOC) [file pone.0056895.s003.doc]

Table S2. *MAT* gene nucleotide polymorphisms in *Sclerotinia sclerotiorum* strains 44Ba1, 44Ba12 and 44Ba18 in comparison to *S. sclerotiorum* strain 1980 (Amselem et al. 2011).

| **Gene name** | **Isolate identifier** | **Gene length** | **Nucleotides at polymorphic positions, bp from 5’-end** | | | | |
| --- | --- | --- | --- | --- | --- | --- | --- |
|  |  |  | **249** | **424** | **856** | **1005** | **-** |
| *MAT1-1-5* | 1980 | 1303 | C | T | A | T |  |
|  | 44Ba1 | 1303 | T | C | G | G |  |
|  | 44Ba12 | 1303 | C | C | G | G |  |
|  | 44Ba18 | 1303 | C | C | G | G |  |
| *MAT1-1-1* |  | | **328** | **367** | **733** | **-** | **-** |
|  | 1980 | 826 | C | C | G |  |  |
|  | 44Ba1 | 1106 | T | T | A |  |  |
|  | 44Ba12 | 354 | T | T^A^ | G^B^ |  |  |
|  | 44Ba18 | 354 | T | T^A^ | G^B^ |  |  |
| *MAT1-2-4* |  | | **61** | **650** | **709** | **778** | **930** |
|  | 1980 | 944 | T | T | A | A | C |
|  | 44Ba1 | 944 | C | G | C | G | T |
|  | 44Ba12 | 944 | T | G | C | G | T |
|  | 44Ba18 | 944 | T | G | C | G | T |
| *MAT1-2-1* |  | | **124** | **1181** | **-** | **-** | **-** |
|  | 1980 | 1289 | C | T |  |  |  |
|  | 44Ba1^C^ | 1289 | A | C |  |  |  |
|  | 44Ba12 | 1289 | A | C |  |  |  |
|  | 44Ba18 | 1289 | A | C |  |  |  |

^A^ Not part of *MAT1-1-1* according to transcript analyses and conceptual translation, see Figures 6, 7, Table 1.

^B^ Not part of *MAT1-1-1*, is on *MAT1-1-1* 3’-end fragment, see Figures 6, 7, Table 1.

^C^ Longest predicted ORF was used, see Figures 6, 7, Table 1.
