# Supplementary material for: The Sclerotinia sclerotiorum Mating Type Locus (MAT) Contains a 3.6-kb Region That Is Inverted in Every Meiotic Generation
Source: PLoS One. 2013 Feb 15;8(2):e56895. doi: 10.1371/journal.pone.0056895 (PMC3574095; doi:10.1371/journal.pone.0056895)
Supplement: Table S3 — Phylogenetic analyses of Sclerotinia sclerotiorum and Botrytis cinerea MAT genes using maximum parsimony. For each analysis, the number of taxa, alignment length, the number of parsimony informative characters, the number and length of most parsimonious trees (MPT) and the consistency (CI) and retention indices (RI), are given. Respective alignments are available as Alignments S6, S7, S8 and S9. (DOC) [file pone.0056895.s004.doc]

Table S3. Phylogenetic analyses of *Sclerotinia sclerotiorum* and *Botrytis cinerea MAT* genes using maximum parsimony. For each analysis, the number of taxa, alignment length, the number of parsimony informative characters, the number and length of most parsimonious trees (MPT) and the consistency (CI) and retention indices (RI), are given. Respective alignments are available as Alignments S6, S7, S8 and S9.

| **Gene** | **Taxa** | **Characters** | **Parsimony informative characters** | **MPTs: Number/Steps** | **CI/RI** |
| --- | --- | --- | --- | --- | --- |
| *MAT1-1-1* | 5 | 1161 | 2 | 4/212 | 0.995/0.5 |
| *MAT1-1-5* | 5 | 1303 | 1 | 1/293 | 1.0/1.0 |
| *MAT1-2-1* | 5 | 1345 | 1 | 1/293 | 1.0/1.0 |
| *MAT1-2-4* | 5 | 977 | 1 | 1/512 | 1.0/1.0 |
