# Supplementary material for: The Sclerotinia sclerotiorum Mating Type Locus (MAT) Contains a 3.6-kb Region That Is Inverted in Every Meiotic Generation
Source: PLoS One. 2013 Feb 15;8(2):e56895. doi: 10.1371/journal.pone.0056895 (PMC3574095; doi:10.1371/journal.pone.0056895)
Supplement: Table S4 — Length comparisons of homologous MAT intergenic spacer regions in Sclerotinia sclerotiorum strains 44Ba1, 44Ba12, 44Ba18 and S. sclerotiorum strain 1980 [17] . (DOC) [file pone.0056895.s005.doc]

Table S4. Length comparisons of homologous *MAT* intergenic spacer regions in *Sclerotinia sclerotiorum* strains 44Ba1, 44Ba12, 44Ba18 and *S. sclerotiorum* strain 1980 (Amselem et al., 2011).

| **Strains/Region** | ***APN2 – MAT1-1-5*** | ***MAT1-1-5 – MAT1-1-1*** | ***MAT1-1-1***^A^ ***– MAT1-2-4*** | ***MAT1-2-4 – MAT1-2-1***^C^ | ***MAT1-2-1 – SLA2***^D^ |
| --- | --- | --- | --- | --- | --- |
| 1980 | 3706 | 406 | 506 | 489 | 779 |
| 44Ba1 | 2897^B^ | 406 | 506 | 500^C^ | 779 |
| 44Ba12 | 2897^B^ | 406 | 506 | 489 | 779 |
| 44Ba18 | 2897^B^ | 406 | 506 | 489 | 779 |

^A^ In Inv+ isolates, *MAT1-1-1* referred to all regions that were homologous to *MAT1-1-1* of Inv- isolates.

^B^ Length differences due to 152 bp and 26 bp deletions at alignment positions 1538 - 1690 and 2237 - 2263, respectively.

^C^ Length difference due to 11 bp insertions at alignment position 56.
