# Supplementary material for: The Sclerotinia sclerotiorum Mating Type Locus (MAT) Contains a 3.6-kb Region That Is Inverted in Every Meiotic Generation
Source: PLoS One. 2013 Feb 15;8(2):e56895. doi: 10.1371/journal.pone.0056895 (PMC3574095; doi:10.1371/journal.pone.0056895)
Supplement: Table S5 — Top blast matches at GenBank against the 250-bp Sclerotinia sclerotiorum motif query using blastn with e-values equal or smaller than 0.33, only a single representative match for each gene in each species is listed. (DOC) [file pone.0056895.s006.doc]

Table S5. Top blast matches at GenBank against the 250-bp *Sclerotinia sclerotiorum* motif query using blastn with e-values equal or smaller than 0.33, only a single representative match for each gene in each species is listed.

| **Species** | **Accession number** | **Gene name** | **Overlap, bp** | **Similarity, %** |
| --- | --- | --- | --- | --- |
| *S. sclerotiorum* | XM_001594147.1 | *MAT1-1-1* | 250 | 100 |
| *Botryotinia fuckeliana* | XN_001546388.1 | *MAT1-1-1* | 201 | 80 |
| *S. sclerotiorum* | XM_001594149.1 | *MAT1-2-1* | 92 | 37 |
| *S. homoeocarpa* | HQ446501.1 | *MAT1-1-1* | 56 | 22 |
| *S. homoeocarpa* | HQ446489.1 | *MAT1-1-1* | 52 | 21 |
