# Supplementary material for: The Sclerotinia sclerotiorum Mating Type Locus (MAT) Contains a 3.6-kb Region That Is Inverted in Every Meiotic Generation
Source: PLoS One. 2013 Feb 15;8(2):e56895. doi: 10.1371/journal.pone.0056895 (PMC3574095; doi:10.1371/journal.pone.0056895)
Supplement: Table S6 — Orientation of MAT inversion region among progeny of four different Sclerotinia sclerotiorum parental strains, 18 to 20 progeny derived from a single apothecium were screened for each parent. For details on parental isolates, see Table S1. (DOCX) [file pone.0056895.s007.docx]

Table S6. Orientation of *MAT* inversion region among progeny of four different *Sclerotinia sclerotiorum* parental strains, 18 to 20 progeny derived from a single apothecium were screened for each parent. For details on parental isolates, see Table S1.

| **Parent** | **Progeny** | **MAT inversion** | **Inv+: Inv- ratio** |
| --- | --- | --- | --- |
| BS011  (Inv-) | BS011sa01 | Inv+ | 11:8 |
|  | BS011sa02 | Inv- |  |
|  | BS011sa03 | Inv- |  |
|  | BS011sa04 | Inv- |  |
|  | BS011sa05 | Inv+ |  |
|  | BS011sa06 | Inv+ |  |
|  | BS011sa07 | Inv+ |  |
|  | BS011sa08 | Inv+ |  |
|  | BS011sa09 | Inv- |  |
|  | BS011sa10 | Inv+ |  |
|  | BS011sa11 | Inv- |  |
|  | BS011sa12 | Inv- |  |
|  | BS011sa14 | Inv+ |  |
|  | BS011sa15 | Inv+ |  |
|  | BS011sa16 | Inv- |  |
|  | BS011sa17 | Inv+ |  |
|  | BS011sa18 | Inv- |  |
|  | BS011sa19 | Inv+ |  |
|  | BS011sa20 | Inv+ |  |
| BS013 (Inv+) | BS013sa01 | Inv- | 13:6 |
|  | BS013sa02 | Inv+ |  |
|  | BS013sa04 | Inv+ |  |
|  | BS013sa05 | Inv+ |  |
|  | BS013sa06 | Inv+ |  |
|  | BS013sa07 | Inv+ |  |
|  | BS013sa08 | Inv+ |  |
|  | BS013sa09 | Inv+ |  |
|  | BS013sa10 | Inv+ |  |
|  | BS013sa11 | Inv+ |  |
|  | BS013sa12 | Inv+ |  |
|  | BS013sa13 | Inv- |  |
|  | BS013sa14 | Inv- |  |
|  | BS013sa15 | Inv- |  |
|  | BS013sa16 | Inv+ |  |
|  | BS013sa17 | Inv- |  |
|  | BS013sa18 | Inv+ |  |
|  | BS013sa19 | Inv- |  |
|  | BS013sa20 | Inv+ |  |
| BS017  (Inv-) | BS017sa01 | Inv- | 7:13 |
|  | BS017sa02 | Inv+ |  |
|  | BS017sa03 | Inv+ |  |
|  | BS017sa04 | Inv- |  |
|  | BS017sa05 | Inv+ |  |
|  | BS017sa06 | Inv- |  |
|  | BS017sa07 | Inv- |  |
|  | BS017sa08 | Inv- |  |
|  | BS017sa09 | Inv- |  |
|  | BS017sa10 | Inv+ |  |
|  | BS017sa11 | Inv- |  |
|  | BS017sa12 | Inv- |  |
|  | BS017sa13 | Inv- |  |
|  | BS017sa14 | Inv- |  |
|  | BS017sa15 | Inv- |  |
|  | BS017sa16 | Inv+ |  |
|  | BS017sa17 | Inv- |  |
|  | BS017sa18 | Inv+ |  |
|  | BS017sa19 | Inv- |  |
|  | BS017sa20 | Inv+ |  |
| BS028  (Inv+) | BS028sa03 | Inv+ | 10:8 |
|  | BS028sa04 | Inv- |  |
|  | BS028sa05 | Inv- |  |
|  | BS028sa06 | Inv+ |  |
|  | BS028sa07 | Inv- |  |
|  | BS028sa08 | Inv+ |  |
|  | BS028sa09 | Inv- |  |
|  | BS028sa10 | Inv+ |  |
|  | BS028sa11 | Inv+ |  |
|  | BS028sa12 | Inv+ |  |
|  | BS028sa13 | Inv- |  |
|  | BS028sa14 | Inv- |  |
|  | BS028sa15 | Inv+ |  |
|  | BS028sa16 | Inv+ |  |
|  | BS028sa17 | Inv- |  |
|  | BS028sa18 | Inv- |  |
|  | BS028sa19 | Inv+ |  |
|  | BS028sa20 | Inv+ |  |
