# Supplementary material for: The Sclerotinia sclerotiorum Mating Type Locus (MAT) Contains a 3.6-kb Region That Is Inverted in Every Meiotic Generation
Source: PLoS One. 2013 Feb 15;8(2):e56895. doi: 10.1371/journal.pone.0056895 (PMC3574095; doi:10.1371/journal.pone.0056895)
Supplement: Table S7 — Formation of apothecial stalks in 38 Sclerotinia sclerotiorum isolates, boldface highlights absence of apothecial stalk formation. (DOC) [file pone.0056895.s008.doc]

Table S7. Formation of apothecial stalks in 38 *Sclerotinia sclerotiorum* isolates, boldface highlights absence of apothecial stalk formation.

| **Strain identifier** | ***MAT* inversion** | **Stalk formation** |
| --- | --- | --- |
| 1B331-1 | Inv+ | + |
| 1B331-2 | Inv+ | + |
| 1B331-5 | Inv+ | + |
| 1B331-6 | Inv+ | + |
| 321B10 | Inv+ | + |
| **321B13** | **Inv+** | **-** |
| 321B17 | Inv+ | + |
| 321B2 | Inv+ | + |
| 321B20 | Inv+ | + |
| 321B21 | Inv+ | + |
| **321B3** | **Inv+** | **-** |
| 321B5 | Inv+ | + |
| 321Db10 | Inv+ | + |
| 321Db15 | Inv+ | + |
| 321Db19 | Inv+ | + |
| 321Db3 | Inv+ | + |
| **321Db4** | **Inv+** | **-** |
| 321Db8 | Inv+ | + |
| 44Ba12 | Inv+ | + |
| **44Ba14** | **Inv+** | **-** |
| **44Ba15** | **Inv+** | **-** |
| **44Ba18** | **Inv+** | **-** |
| 44Ba4 | Inv+ | + |
| 44Bb11 | Inv+ | + |
| 44Bb13 | Inv+ | + |
| 44Bb14 | Inv+ | + |
| 44Bb15 | Inv+ | + |
| **44Bb20** | **Inv+** | **-** |
| 44Bb21 | Inv+ | + |
| 1B331-3 | Inv- | + |
| 1B331-4 | Inv- | + |
| 1B331-7 | Inv- | + |
| 1B331-8 | Inv- | + |
| 321Db18 | Inv- | + |
| 321Db5 | Inv- | + |
| 44Ba1 | Inv- | + |
| 44Ba13 | Inv- | + |
| 44Ba2 | Inv- | + |
