# Supplementary material for: The Sclerotinia sclerotiorum Mating Type Locus (MAT) Contains a 3.6-kb Region That Is Inverted in Every Meiotic Generation
Source: PLoS One. 2013 Feb 15;8(2):e56895. doi: 10.1371/journal.pone.0056895 (PMC3574095; doi:10.1371/journal.pone.0056895)
Supplement: Table S8 — Primers used for PCR and sequencing of the Sclerotinia sclerotiorum mating type locus in isolates 44Ba1, 44Ba12 and 44Ba18. The last letter in a primer name indicates the primer direction, forward and reverse, respectively. (DOC) [file pone.0056895.s009.doc]

Table S8. Primers used for PCR and sequencing of the *Sclerotinia sclerotiorum* mating type locus in isolates 44Ba1, 44Ba12 and 44Ba18. The last letter in a primer name indicates the primer direction, forward and reverse, respectively.

| **Primer name** | **Primer DNA sequence (5’ → 3’)** |
| --- | --- |
| MAT_1069F | CAGAATTTGATCGACGTCAGAG |
| MAT_1887F | GCTGAGGCTGGAAATAACGCA |
| MAT_2703F | AACAAGCTCATGAGTATATTCATA |
| MAT_3466R | CTTAACCTACAACTACCAACCA |
| MAT_4417F | GCTTAGAACACTTTCATATAAT |
| MAT_4584F | ATATCAATATCATCAGCAAAATA |
| MAT_4694F | TGAATATACTCCAGCCACACAGCT |
| MAT_5298F | ATTTACACAATGCCGGAGGCTTA |
| MAT_5798R | CGGACATGAAGCAGGTTCTCAT |
| MAT_5985R | CTTCACGTCGCATTTGTATGGC |
| MAT_6405F | GCTTCTTCAATAATTCTCTTCCTA |
| MAT_6555R | TTTGACACACGATACTGAGTAGTTGT |
| MAT_6651R | TGTTGTATACCACCCTCAATTTC |
| MAT_6707R | TTACTACGGAACAATATATATG |
| MAT_6788R | CATATAGAAGGCTAGGTAGATGT |
| MAT_6813R | GAATATTTCCAAGCACATTCAAAATC |
| MAT_7049R | ACGAAGACAAACAATCCCTTCTTCAT |
| MAT_7072F | GTTCGTTTCCAGCACATATC |
| MAT_11574F | TGGAGATATCCGAGTGGAAGAAG |
| MAT_11655F | GCCCTCCCTACAAGTGTCATCGT |
| MAT_11662F | CTACAAGTGTCATCGTCCTCAAT |
| MAT_11899R | AGGTCGAAATCCTACAACTTGAG |
| MAT_11930R | CAGGTAAGTTTCCTTGAAGGGATT |
| MAT_12530F | TGCATCAGCAGCCTTGGAGAGCTCGT |
| MAT_12754R | CAGTCTACTGTCACATTTTTCCGT |
| MAT_13451R | GGTCGAAAAGCTTAAACGCGAG |
| MAT_13589R | AGCTCAGGAGGCCATTGACAAGC |
| MAT_14587R | TTCAGATCTTATGACTCTACAG |
