# Supplementary material for: The Sclerotinia sclerotiorum Mating Type Locus (MAT) Contains a 3.6-kb Region That Is Inverted in Every Meiotic Generation
Source: PLoS One. 2013 Feb 15;8(2):e56895. doi: 10.1371/journal.pone.0056895 (PMC3574095; doi:10.1371/journal.pone.0056895)
Supplement: Table S9 — Sequencing primers targeting the MAT inversion in Sclerotinia sclerotiorum strains 44Ba12 and 44Ba18. The last letter in a primer name indicates the primer direction, forward and reverse, respectively. (DOC) [file pone.0056895.s010.doc]

Table S9. Sequencing primers targeting the *MAT* inversion in *Sclerotinia sclerotiorum* strains 44Ba12 and 44Ba18. The last letter in a primer name indicates the primer direction, forward and reverse, respectively.

| **Primer name** | **Primer DNA sequence (5’ → 3’)** |
| --- | --- |
| INV_547F | ATCAGCACGATAAAGAATCCA |
| INV_1286R | CATAATAAGCTGGAGTACTTGT |
| INV_1923R | TCCGAAGTTGCCTTTCATCTC |
| INV_2359F | GAGGCACATATAGAGGCATAT |
| INV_2746F | GTGTCACGAAAGATTCGGGAATA |
