# Supplementary material for: The Sclerotinia sclerotiorum Mating Type Locus (MAT) Contains a 3.6-kb Region That Is Inverted in Every Meiotic Generation
Source: PLoS One. 2013 Feb 15;8(2):e56895. doi: 10.1371/journal.pone.0056895 (PMC3574095; doi:10.1371/journal.pone.0056895)
Supplement: Table S10 — Sequencing primers used in Sclerotinia sclerotiorum strain 44Ba1 targeting the MAT inversion region in S. sclerotiorum strains 44Ba12 and 44Ba18. The last letter in a primer name indicates the primer direction, forward and reverse, respectively. (DOC) [file pone.0056895.s011.doc]

Table S10. Sequencing primers used in *Sclerotinia sclerotiorum* strain 44Ba1 targeting the *MAT* inversion region in *S. sclerotiorum* strains 44Ba12 and 44Ba18. The last letter in a primer name indicates the primer direction, forward and reverse, respectively.

| **Primer name** | **Primer DNA sequence (5’ → 3’)** |
| --- | --- |
| MAT_7232F | CCCTGTTACACAACACATTGCTTCCACT |
| MAT_7303R | ACAATGACACCTTTAGGCATCTC |
| MAT_7540R | GGTGGAGAGATATCGCCAGGAACAT |
| MAT_7776R | ATTGGTGGATAAATGTCCCAGGTT |
| MAT_8003F | TATCTCATGATGGAATAAAAATAT |
| MAT_8077R | CATTGAGCATGGATCATGAAAGAAGA |
| MAT_8209R | ACTCTTGGAAAGAGAAGATTATA |
| MAT_8453R | GCAATGTATTTGCAGACGAGATA |
| MAT_8750R | TGCGAATAGCTTTATGAAACTCGA |
| MAT_8905F | AGCAATTCCTAAAAATGGTTGTG |
| MAT_9538F | TCACACTTAGAGGATGTCTCTGTC |
| MAT_9040R | AATGCAGGTGCCGAGACATCGA |
| MAT_8873F | CACCGTGTAATTAACGTCAT |
| MAT_8917F | ATGGTTGTGGAGTTGTCGTCTTG |
