# Supplementary material for: The Sclerotinia sclerotiorum Mating Type Locus (MAT) Contains a 3.6-kb Region That Is Inverted in Every Meiotic Generation
Source: PLoS One. 2013 Feb 15;8(2):e56895. doi: 10.1371/journal.pone.0056895 (PMC3574095; doi:10.1371/journal.pone.0056895)
Supplement: Table S11 — Primer pairs used for Sclerotinia sclerotiorum Inv+ PCR screening, and PCR amplification and DNA sequencing of inversion breakpoints and MAT inversion region. The ‘f’ or ‘r’ in a primer name indicates the primer direction, forward and reverse, respectively. (DOC) [file pone.0056895.s012.doc]

Table S11. Primer pairs used for *Sclerotinia sclerotiorum* Inv+ PCR screening, and PCR amplification and DNA sequencing of inversion breakpoints and *MAT* inversion region. The ‘f’ or ‘r’ in a primer name indicates the primer direction, forward and reverse, respectively.

| **Primer name** | **Primer DNA sequence (5’→ 3’)** |
| --- | --- |
| SS1f | GTCTGCTATCCTCAGTGTCTTATGG |
| SS1r2 | CTCTTCCATCAGCTTTCCTATTGC |
| SS2f2 | ACCATATGCATTCTGAGTGGAAGC |
| SS2r | ACTCCCCACTAATCTACACTTTCGG |
| SSf2 | CTCACTCTCGTTGCGTATCTCTT |
| SSf3 | TCGTCTAACATGCAATTACAGGCAAC |
| SSf5 | TTCCAAGAGTCATAATTCAAAGG |
| SSf6 | CCTTGGTATTGGCACACCTGAACG |
| SSr1 | AGGGCAATGTATTTGCAGACGAG |
| SSr2 | GGCAAGAAATATTCTGCTGACATAGG |
| SSr4 | GCAGCCGATTTGGGGCTGGT |
| SSr5 | TGCAGTCGACATCACACCTGTCG |
| SSr7 | ACACTCTATCTACACCACTGCC |
| Type-IIF | CCGTTTAAGGGAAATCCAGA |
| Type-IIR | ACGTGCATCCAAGAAGACGC |
