# Supplementary material for: The Sclerotinia sclerotiorum Mating Type Locus (MAT) Contains a 3.6-kb Region That Is Inverted in Every Meiotic Generation
Source: PLoS One. 2013 Feb 15;8(2):e56895. doi: 10.1371/journal.pone.0056895 (PMC3574095; doi:10.1371/journal.pone.0056895)
Supplement: Table S12 — Primers used for Sclerotinia sclerotiorum MAT gene expression analysis. (DOC) [file pone.0056895.s013.doc]

Table S12. Primers used for *Sclerotinia sclerotiorum MAT* gene expression analysis.

| **Gene** | **Primer** | **Primer Sequence (5’→ 3’)** |
| --- | --- | --- |
| *MAT1-1-1* | MAT1F | ATACAGCCACTTACCTACCATACAGC |
|  | MAT1R | CACTGAGGATGGCAGACT |
| *MAT1-2-1* | MAT2F | ATGTCTCTGTCCACCCTCAGACTTGG |
|  | MAT2R | TCAGATATCCGTTCGTTTCCAGCACAT |
| *MAT1-1-5* | MAT5F | AGCTTTCTTGAGGAGGAAGTATTTA |
|  | MAT5R | GACGAGGGCATCTAGAAATGCAG |
| *MAT1-2-4* | MAT4F | GTCTCAGATCAACGCAATGTA |
|  | MAT4R | TGGTGCTGACCCTTGTTTGTCG |
| *MAT1-1-1* 3’-end fragment | FusionF | GTATATACATCCATCCAACTCCTA |
|  | FusionR | ACATATCGCTAGAACTACCTTGA |
| *Actin* | F | TGGTTCTGGAATGTGTAAGGCC |
|  | R | TGTAGAAAGTGTGATGCCAGAT |
